# Evolutionary Divergence of the C-terminal Domain of Complexin Accounts for Functional Disparities between Vertebrate and Invertebrate Complexins

**DOI:** 10.3389/fnmol.2017.00146

**Published:** 2017-05-26

**Authors:** Rachel T. Wragg, Daniel A. Parisotto, Zhenlong Li, Mayu S. Terakawa, David Snead, Ishani Basu, Harel Weinstein, David Eliezer, Jeremy S. Dittman

**Affiliations:** ^1^Department of Biochemistry, Weill Cornell Medical College, New YorkNY, United States; ^2^Department of Physiology and Biophysics, Weill Cornell Medical College, New YorkNY, United States; ^3^Institute for Computational Biomedicine, Weill Cornell Medical College, New YorkNY, United States

**Keywords:** complexin, membrane binding, synaptic transmission, synaptic vesicles, molecular dynamics, evolutionary conservation and diversification, SNAREs, *C. elegans*

## Abstract

Complexin is a critical presynaptic protein that regulates both spontaneous and calcium-triggered neurotransmitter release in all synapses. Although the SNARE-binding central helix of complexin is highly conserved and required for all known complexin functions, the remainder of the protein has profoundly diverged across the animal kingdom. Striking disparities in complexin inhibitory activity are observed between vertebrate and invertebrate complexins but little is known about the source of these differences or their relevance to the underlying mechanism of complexin regulation. We found that mouse complexin 1 (mCpx1) failed to inhibit neurotransmitter secretion in *Caenorhabditis elegans* neuromuscular junctions lacking the worm complexin 1 (CPX-1). This lack of inhibition stemmed from differences in the C-terminal domain (CTD) of mCpx1. Previous studies revealed that the CTD selectively binds to highly curved membranes and directs complexin to synaptic vesicles. Although mouse and worm complexin have similar lipid binding affinity, their last few amino acids differ in both hydrophobicity and in lipid binding conformation, and these differences strongly impacted CPX-1 inhibitory function. Moreover, function was not maintained if a critical amphipathic helix in the worm CPX-1 CTD was replaced with the corresponding mCpx1 amphipathic helix. Invertebrate complexins generally shared more C-terminal similarity with vertebrate complexin 3 and 4 isoforms, and the amphipathic region of mouse complexin 3 significantly restored inhibitory function to worm CPX-1. We hypothesize that the CTD of complexin is essential in conferring an inhibitory function to complexin, and that this inhibitory activity has been attenuated in the vertebrate complexin 1 and 2 isoforms. Thus, evolutionary changes in the complexin CTD differentially shape its synaptic role across phylogeny.

## Introduction

Precise synaptic transmission is critical for proper nervous system function, and over the past 25 years, most of the proteins required for this process have been identified and characterized. A mechanistic picture has emerged based on the assembly of SNARE proteins residing on the synaptic vesicle (SV) and plasma membrane (Sollner and Rothman, 1994). This assembly is tightly orchestrated by a set of conserved proteins including Munc13, Munc18, and synaptotagmin (Sudhof, 2013; Rizo and Xu, 2015). Although the complete molecular picture of the events controlling SV fusion is far from fully developed, there is general agreement on the impact of perturbing the SNAREs, Munc13, Munc18, and synaptotagmin in many highly divergent experimental preparations such as the squid giant synapse, worm and fly neuromuscular junctions, as well as rodent cultured neurons and acute rodent brain slices (Augustine et al., 1996; Weimer and Jorgensen, 2003; Sudhof and Rothman, 2009; Kochubey et al., 2011; Sudhof and Rizo, 2011). Consensus of both sequence and function across such a broad range of synapses implies a deep mechanistic conservation of calcium-regulated secretion in all animals, consistent with the assertion that there is a single overarching molecular pathway for SV fusion in neurons shared across phylogeny. However, another key SNARE-binding protein has proven more difficult to fit into this picture. Complexin is a small (130–150 residue) cytoplasmic protein that binds directly to the assembled SNARE complex via a highly conserved alpha helical domain termed the central helix (CH) (McMahon et al., 1995; Melia, 2007; Brose, 2008; Trimbuch and Rosenmund, 2016). Human complexin mutations (*CPLX1* gene) are associated with severe epilepsy, cortical atrophy, and intellectual disability (Karaca et al., 2015; Redler et al., 2017). Loss-of-function studies in different model synapses revealed similarities as well as prominent differences in complexin function. For instance, while almost all studies agree that loss of complexin leads to a decrease in calcium-triggered exocytosis, the regulation of spontaneous fusion by complexin appears to have diverged between vertebrates and invertebrates (Trimbuch and Rosenmund, 2016). SV fusion in the absence of calcium influx (spontaneous fusion) is either decreased or slightly increased in several mammalian synapses lacking complexin depending on the preparation and the details of complexin removal (Xue et al., 2007; Maximov et al., 2009; Strenzke et al., 2009; Lin et al., 2013; Yang et al., 2013). In contrast, spontaneous SV fusion is highly elevated (between 10- and 20-fold) in worm and fly synapses lacking complexin (Huntwork and Littleton, 2007; Hobson et al., 2011; Martin et al., 2011). Interestingly, vertebrate complexin 3/4 isoforms have been proposed to inhibit spontaneous release in retinal bipolar cell synapses (Vaithianathan et al., 2013, 2015), suggesting a functional divergence between complexin isoforms within the vertebrate subphylum. Another recent study found that the rate of SV fusion in the calyx of Held is transiently elevated by a factor of more than 10-fold in the absence of mCpx1, but only for a brief time lasting a few 100 ms after SVs initially dock and prime following a previous SV fusion event (Chang et al., 2015). These observations hint at a transient role for mammalian complexin 1 in preventing premature fusion during the process of docking and priming, whereas invertebrate complexins are constitutively required to inhibit spontaneous fusion.

Relative to the other core SV fusion machinery, complexin is a poorly conserved protein. The 25 residues defining the CH constitute the only extensive region of complexin exhibiting strong conservation between phyla. This CH domain mediates a direct SNARE interaction and is required for all known complexin function in both vertebrate and invertebrate synapses (Giraudo et al., 2006; Xue et al., 2007; Maximov et al., 2009; Cho et al., 2010; Martin et al., 2011; Yang et al., 2015). Three additional regions of complexin have been defined both structurally and functionally: the N-terminal domain (NTD), the accessory helix domain (AH), and the C-terminal domain (CTD) comprising the latter half of complexin (Trimbuch and Rosenmund, 2016). The NTD serves a positive function in regulating fusion whereas the AH and CTD contribute to an inhibitory activity of complexin (Xue et al., 2007, 2010; Kummel et al., 2011; Martin et al., 2011; Kaeser-Woo et al., 2012; Buhl et al., 2013; Iyer et al., 2013; Wragg et al., 2013; Cho et al., 2014; Lai et al., 2014; Radoff et al., 2014). How does a protein domain with little or no primary sequence homology share a conserved function? The complexin CTD lacks meaningful sequence identity between phyla but a common motif predicted in all known complexin CTD sequences is an amphipathic helix region near the end of the protein (Seiler et al., 2009; Wragg et al., 2013; Snead et al., 2014; Gong et al., 2016). Several recent studies have proposed that the amphipathic region of the CTD mediates a curvature-sensitive membrane binding interaction that directs both mammalian and nematode complexin to SVs (Wragg et al., 2013; Snead et al., 2014; Gong et al., 2016). Without the CTD, the inhibitory function of complexin is impaired (Xue et al., 2009; Kaeser-Woo et al., 2012; Wragg et al., 2013), as is complexin localization at the synapse (Buhl et al., 2013; Iyer et al., 2013; Wragg et al., 2015). In addition to the amphipathic region, the CTD of all complexins terminates with either a second hydrophobic lipid-binding motif or a lipidated CAAX box motif, further emphasizing a potential membrane-interacting role for this region of complexin (Reim et al., 2005; Cho et al., 2010; Buhl et al., 2013; Iyer et al., 2013). Despite these conserved membrane-interacting features, several studies have described a range of imperfect functional rescue between species when exchanging mouse and fly complexins (Xue et al., 2009; Cho et al., 2010). Is the CTD functionally conserved despite the wide variety of primary sequences across phyla? Do the differences in CTD sequences account for the functional differences between vertebrate and invertebrate complexins? We systematically investigated nematode and mammalian complexin 1 orthologs using a combination of *in vitro, in vivo*, and computational approaches and found that differences in the CTD account for divergence of complexin inhibitory function. Moreover, these differences are not simply due to large variations in membrane binding. We propose that other divergent protein interactions within the CTD account for functional differences in complexin across phylogeny.

## Materials and Methods

### Animals

*Caenorhabditis elegans* were maintained on agar nematode growth media (NGM) at 20°C and seeded with OP50 bacteria as previously described (Brenner, 1974). Strains employed in this study are listed in Supplementary Table S3. Robust synaptic expression of all arrays was verified by measuring synaptic fluorescence to check expression levels against those that can fully rescue complexin mutants as previously described (Martin et al., 2011; Wragg et al., 2013).

### Pharmacological Assays

To measure aldicarb sensitivity, 20–25 young adult animals were placed on agar plates containing 1 mM aldicarb (Chem Services) and scored for paralysis at 10 min intervals for 2 h. Each genotype was tested 8–10 times and paralysis curves were generated by averaging paralysis time courses for each plate as described previously (Dittman and Kaplan, 2008; Martin et al., 2011; Wragg et al., 2013). Percent rescue based on *t*_0.5_ was calculated by first interpolating the time at which 50% of the worms paralyzed for each trial, averaging the single-trial *t*_0.5_ values together, and normalizing to wild type (100%) *t*_0.5_ and *cpx-1* (0%) *t*_0.5_ values according to **Equation 1**.

(1) $$\begin{array}{c} \%\;Rescue\left[Strain\right]=100\cdot \left(t_{0.5}\left[Strain\right]-t_{0.5}\left[cpx\right]\right)/t_{0.5}\left[WT\right]\;\left(1\right) \end{array}$$

### Steady-State Fluorescence Imaging and Quantification

To measure protein expression levels, animals were immobilized using 2,3-butanedione monoxime (Alfa Aesar) (30 mg/mL) mounted on 2% agarose pads. An inverted Olympus microscope (IX81), using a laser scanning confocal imaging system (Olympus Fluoview FV1000 with dual confocal scan heads) and an Olympus PlanApo 60X 1.42 NA objective was used. Rescuing complexin constructs were C-terminally tagged with GFP separated by a 12 residue linker (GGSGGSGGSAAA). Synaptic protein levels were estimated by measuring background-subtracted fluorescence within dorsal cord varicosities. A fluorescent slide was imaged to monitor laser stability over time and the dorsal cord axonal fluorescence was normalized to the slide value for all measurements. For the data plotted in **Figure 3F**, the normalized axonal fluorescence for all three strains was normalized to the worm CPX-1:: GFP strain for comparison. Data were analyzed with custom software in IGOR Pro (WaveMetrics, Lake Oswego, OR, United States) (Burbea et al., 2002; Dittman and Kaplan, 2006). As previously reported, we did not observe a correlation between expression levels and rescue efficiency (Wragg et al., 2013; Radoff et al., 2014). The single-copy CPX-1:: GFP transgene fully rescued in all behavioral assays even though it was expressed near the lower limit of our imaging sensitivity. All transgenic strains used in this study displayed a higher expression level of CPX relative to this single-copy strain.

### Protein Purification

CPX-1+W (JP767), CPX-1 (ΔCT)+W (JP773), mCpx1+W (JP790), mCpx1 (ΔCT)+W (JP791), CPX-1(FFF/AAA)+W (JP793), CPX-1 (FFF/III)+W (JP794), CPX-1 (LV/EE)+W (JP915), CPX-1(Δ6)+W (JP916), CPX-1 (Δ6mouse7)+W (JP917) constructs were cloned into the pET28a vector using standard techniques. These constructs contain a His_6_ tag, a T7 tag and a thrombin cleavage site to facilitate purification. BL21-DE3 *Escherichia coli* were transformed and grown in Luria-Bertani media (LB) with kanamycin (50 μg/mL) to an optical density of 0.6. Cells were induced with isopropyl thiogalactopyranoside (IPTG) (400 μg/ml), grown for 3 h at 37°C, pelleted, resuspended in buffer (350 mM NaCl, 20 mM imidazole, 20 mM Tris–HCl pH 8, 1.5 mM BME, 2 mM DTT), lysed by sonication, and pelleted at 40,000 r.p.m. for 40 min. The supernatant was purified on a Ni-NTA column (Qiagen, Hilden, Germany). Protein was eluted in elution buffer (350 mM NaCl, 250 mM Imidazole, 20 mM Tris–HCl, 1.5 mM BME, 2 mM DTT) then dialyzed into buffer (150 mM NaCl, 50 mM Tris–HCl pH 8). Protein was then concentrated and FPLC was performed. Sephadex G-25 Fine beads (Sigma) were then used for buffer exchange (150 mM NaCl, 50 mM Tris–HCl pH 8, 5 mM EGTA). Protein concentrations were estimated by absorbance at 280 nm using the extinction coefficient. For the mouse mCpx1 NMR studies, the CTD construct (residues 71–134) was cloned into a SUMO fusion vector and expressed and purified as previously described for worm complexin using nickel-affinity chromatography. Briefly, BL21(DE3) *E. coli* cells were transformed with the relevant plasmid, grown in LB media for 4 h as a small culture and transferred to 100 mL minimal media containing ^15^N-labeled ammonium chloride and ^13^C-labeled glucose and grown overnight. Cells were grown in 1 L minimal media to an optical density of 0.6 before induction with IPTG for 3–4 h. Cells were lysed by sonication on ice, supernatants were clarified by centrifugation at 40,000 r.p.m. for 45 min. The SUMO-tagged fusion protein was purified from supernatants on a Ni-NTA column and dialyzed into 20 mM Tris pH 8, 150 mM NaCl, 1 mM dithiothreitol, followed by cleavage of the SUMO tag using the SUMO protease Ulp1. A second Ni-NTA affinity purification was used to remove the SUMO tag. Proteins were then dialyzed into distilled water, frozen and lyophilized. For NMR, lyophilized proteins were dissolved in 50 mM phosphate pH 6.1, 1 mM dithiothreitol, 0.5 mM EDTA, with 60 mM NaCl. Protein concentrations were estimated by absorbance at 280 nm using the coefficients of the individual amino acids in the protein sequence.

### Small Unilamellar Vesicle (SUV) Preparation

Lipids were obtained from Avanti Polar Lipids and stored at -20°C. A lipid mixture composed of 85% 1-palmitoyl-2-oleoyl-phosphatidylcholine (POPC), and 15% 1-palmitoyl-2-oleoyl-phosphatidylserine (POPS) was dried under a stream of N_2_ gas then residual solvent was removed under vacuum for 2 h. The lipid film was then rehydrated in assay buffer (150 mM NaCl, 50 mM Tris–HCl pH8, 5 mM EGTA) to obtain a lipid concentration of 4 mM. The resulting SUVs underwent bath sonication and pelleted at 60,000 r.p.m. for 2 h (Sorvall RC M120 EX Ultracentrifuge, S120AT2 rotor). Vesicle size and purity were verified by dynamic light scattering using a Zetasizer Nano-S (Malvern Instruments). Lipid concentration was determined based on the amounts of starting lipid and using a phosphate quantification assay. Perchloric acid was added to lipid samples and heated to 150°C for 1 h. Ammonium molybdate and ascorbic acid were added to samples and heated for 10 min at 100°C. Absorbance was measured at 797 nm and lipid concentrations were obtained through comparison to phosphate standards. Vesicles were stored at 4°C and used within 1 week.

### Fluorescence Titration Measurements

Tryptophan fluorescence was measured at 22°C with either a spectrofluorometer (Photon Technology International) or a SpectraMax M5 microplate reader (Tecan). For the spectrofluorometer, emission spectra were recorded between 300 and 450 nm (1 nm step) with an excitation wavelength of 280 nm, at slit widths of 4 nm. For the plate reader, emission at 350 nm was recorded in a 96-well plate using an excitation wavelength of 280 nm with 6 flashes per read. Protein–lipid binding was determined from the increase in tryptophan emission fluorescence intensity upon addition of SUVs corrected for fluorescence in SUVs alone. The data were analyzed using custom software in IGOR Pro (WaveMetrics, Lake Oswego, OR, United States).

### NMR Spectroscopy

Perdeuterated CTD and perdeuterated DPC (Avanti Polar Lipids) were used for triple resonance experiments used to assign the CTD in the presence of DPC (i.e., ^15^N, ^13^C, ^2^H labeled protein) and for the HSQC-NOESY-HSQC experiment for the CTD with DPC micelles (i.e., ^15^N, ^2^H labeled protein). DPC micelles were prepared by resuspending a dried lipid film at the desired stock concentration (Snead et al., 2017). Experiments included TROSY versions of HNCACB, HN(CO)CACB, HNCACO, HNCO, HNCA, and HNCOCANH. Data were collected on 600 MHz (Weill Cornell) and 900 MHz (New York Structural Biology Center) cryoprobe-equipped spectrometers and indirectly referenced to 4,4-dimethyl-4-silapentane-1-sulfonic acid and ammonia based on the position of the water resonance. Cα-Cβ secondary shifts were calculated as the difference between the observed carbon chemical shifts and random coil values tabulated from linear hexapeptides in 1M urea at pH 5.0 and 25°C.

### Calculation of Amphipathic Moments and Helicity

The amphipathic moment vector was defined by **Equation 2** where 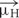 is the net moment vector of an N-residue helix (in complex notation), *r*_k_ = hydrophobicity of the *k*^th^ residue using the Moon-Fleming scale (multiplied by -1) and δ = 100° is the angle between successive residue side chains moving counter-clockwise (Eisenberg et al., 1982; Moon and Fleming, 2011).

(2) $$\begin{array}{c} \vec{\mu_{H}}=\sum_{k=1}^{N}r_{k}\{\cos\left(\left(k-1\right)\cdot \delta \right)-i\cdot \sin\left(\left(k-1\right)\cdot \delta \right) \end{array}$$

The distribution of amphipathic moment magnitudes for random 12-mer and 18-mer peptides was estimated by generating 10^6^ random peptide sequences (excluding proline from all but the first two and last two residues) and computing the amphipathic moment for each peptide. The proline-free constraint was implemented to allow for stable alpha helix packing. The cumulative distributions of these ensembles are shown in **Figure 9G**. Because the aspartate and glutamate hydrophobicity were assigned at low pH in the Moon-Fleming scale, we substituted those values with the octanol hydrophobicity values (-3.64 and -3.63 kCal/mol, respectively). Percent helicity was computed using Agadir as described previously (Radoff et al., 2014), and average values were normalized to the average nematode helicity for comparison.

### Molecular Dynamics Simulations

The peptide-membrane binding free energy profiles (potentials of mean force, PMF) were computed along the normal of a model lipid bilayer, using Molecular Dynamics (MD) simulations with the CHARMM36 all-atom force field (MacKerell et al., 1998). The lipid bilayer was modeled by a compositionally symmetric mixture of 100 DOPC:DOPE:DOPS lipids (mole ratio of 60:25:15) pre-assembled using the CHARMM-GUI server (Jo et al., 2008). The bilayer surfaces were aligned parallel to the XY plane and solvated in a cubic water box (70 Å × 70 Å × 110 Å) with periodic boundary conditions (PBCs). Two complexin peptides were positioned near the bilayer (one on each side) to exploit available symmetry. Both peptides were modeled as initially disordered (Snead et al., 2014), and both ends of each peptide were capped with neutral end groups (acetylated N-terminus and amidated C-terminus). In addition, each system was brought to electrical neutrality and adjusted to a NaCl concentration of 0.15 M by randomly replacing water molecules with ions. The equilibration phase of the simulations was conducted with the NAMD software (version 2.10) under isothermal-isobaric (NPT) ensemble (*P* = 1 atmosphere, *T* = 310 K) (Phillips et al., 2005). A 2000-step energy minimization and 2 nanoseconds (ns) MD simulation with harmonic restraints (force constant *k* = 5 kcal/mol) were conducted on the positions of both the lipid heavy atoms and peptide backbone atoms using an integration time interval of 1 femtosecond (fs). The system was further equilibrated for another 10 ns with an integration time interval of 2 fs after removal of restraints on the lipid heavy atoms. In all simulations, the particle mesh Ewald (PME) algorithm was used for long-range electrostatic interactions, while a 14-Å cutoff distance was used for van der Waals interactions (Darden et al., 1993).

### Free Energy Calculation

After equilibration, the bilayer-binding PMF of each peptide was explored using united free energy dynamics (UFED) (Cuendet and Tuckerman, 2014), an enhanced sampling approach that combines the advantages of driven adiabatic free energy dynamics (dAFED) and metadynamics methods (Laio and Parrinello, 2002; Zhu et al., 2002). Within the framework of dAFED, the vertical peptide-bilayer separation distance, Δ*z* was chosen as a collective variable (CV) for each peptide and computed during the simulations. The Δ*z* is defined as the distance between the center of mass (COM) of sidechain heavy atoms of the four residues in the middle (residues 138–141 for worm complexin C-terminal peptide) and the COM of all lipid phosphate atoms along the bilayer normal. To ensure the system quickly crosses large free energy barriers, Δ*z* was harmonically (*k* = 50 kcal/mol/Å) coupled to a fictitious particle. By choosing a much larger virtual mass for the fictitious particle (2 × 10^11^ kJ/mol/Å^2^) than the total mass of the physical system, the dynamics of the fictitious particle was adiabatically decoupled from the physical system. This allowed assignment of a high temperature (*T* = 2500 K) to the dynamics of the fictitious particle that is able to cross high free energy barriers and drive the physical system to evolve faster along Δ*z* at room temperature. An external biasing Gaussian potential of fixed height (0.1 kcal/mol) and width (0.1 Å) was added to the Hamiltonian of the system every 2500 time steps as a history-dependent function of Δ*z*. All UFED simulations were conducted using the ACEMD molecular dynamics software (Harvey et al., 2009) with the pluMED software as a plugin that supplies the UFED function (Abrams and Tuckerman, 2008; Bonomi et al., 2009). With the same atomic coordinates from the final configuration of the equilibration run, the atomic velocities were randomly regenerated at 310 K to start 10 independent replicas of UFED simulations that sample the one-dimensional free energy space in a parallel manner. The upper limit of Δ*z* was set to 30.0 Å to avoid interactions between the two peptides. By adopting the hydrogen mass repartitioning scheme, we conducted all UFED simulations with a time step of 4 fs under canonical (NVT) ensemble (*T* = 310 K) (Bonomi et al., 2009). For each system, the total sampling time exceeded 3 μS, and the time series of Δ*z* and its corresponding virtual counterpart (i.e., the trajectory of the fictitious particle) were used to reconstruct the PMF along Δ*z* based on the thermodynamic forces (Cuendet and Tuckerman, 2014).

### Statistics and Protein Sequence Analysis

For single comparisons, statistical significance was defined as *p* < 0.01 by Student’s *t*-test. In cases where multiple comparisons were made using the same data sets, ANOVA followed by the *post hoc* Tukey–Kramer method was used to compute significance as defined by *p* < 0.01. Multiple protein sequence alignment was performed using Clustal Omega. Helical wheels were generated using custom software implemented in Igor Pro. Helical propensity was computed with Agadir (Munoz and Serrano, 1997).

## Results

### Conserved Features of Complexin–SNARE Interactions

**FIGURE 1:**
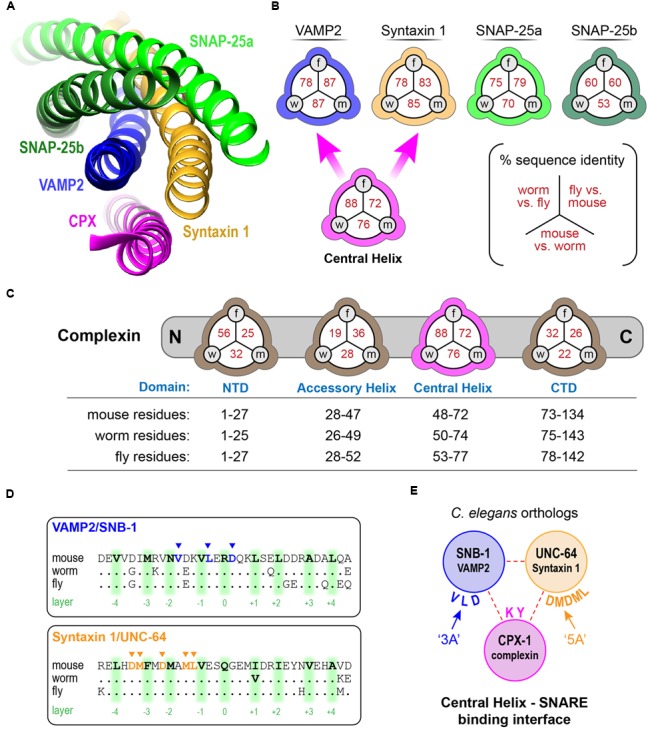
**Conserved features of complexin–SNARE interactions. (A)** Cartoon of the central helix of complexin 1 bound to the ternary SNARE complex as indicated. Based on PDB1KIL (Chen et al., 2002). **(B)** Pair-wise primary sequence identity of the four neuronal SNARE domains between *C. elegans* (*w*), *D. melanogaster* (*f*), and mouse (*m*). Also indicated, sequence identity of the complexin 1 central helix (*magenta*) that binds directly to VAMP2 and Syntaxin 1. **(C)** The four protein domains of complexin are shown with their associated pair-wise protein sequence identity for worm, fly, and mouse as in **(B)**. The specific residues used to define the domains are listed for each species below. **(D)** 33 resides of the SNARE domains for VAMP2 (*Top*) and Syntaxin 1 (*Bottom*) are aligned for mouse, worm, and fly with 9 central SNARE layer residues highlighted in green. The residues that directly interact with complexin are depicted in blue and orange (*arrowheads*). Note that all 8 of these residues are conserved between the three species. **(E)** The *C. elegans* orthologs of the neuronal SNAREs. Key residues that contribute to the binding reaction are mutated to alanines in the ‘3A’ *snb-1* VAMP2 mutant and ‘5A’ *unc-64* Syntaxin 1 mutant.

**FIGURE 2:**
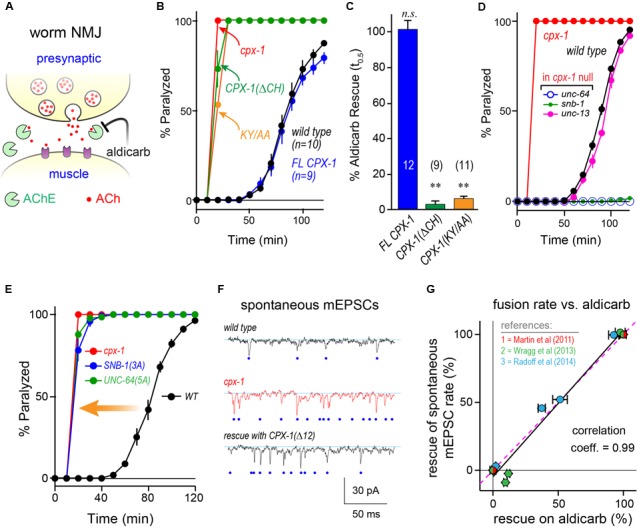
**Function impact of disrupting complexin–SNARE interactions. (A)** Cartoon of the worm neuromuscular junction (NMJ) depicting acetylcholine (ACh, red) release as well as synaptic cleft cholinesterase (AChE, *green*), and the cholinesterase inhibitor aldicarb. **(B)** Average paralysis time course on 1 mM aldicarb for wild type (*black*), *cpx-1(ok1552)* null mutant (*red*), full-length rescue CPX-1 (*blue*), and rescue with CPX-1 either lacking its central helix (ΔCH, *green*), or with two alanine substitutions in two conserved central helix residues (KY/AA, *orange*) to disrupt SNARE binding. All rescue strains are in *cpx-1(ok1552)* null mutant background. **(C)** Quantification of aldicarb rescue based on the time to 50% paralysis (*t*_0.5_) for full-length CPX-1 (*blue*), central helix deletion (ΔCH, *green*), and the central helix double point mutant (KY/AA, *orange*). **(D)** Aldicarb paralysis time course for wild type (*black*), *cpx-1* (*red*), or *cpx-1* double mutant together with a hypomorphic mutant of either *unc-64(e246)* Syntaxin 1 (*blue*), *snb-1(md247)* Synaptobrevin 1 (*green*), or *unc-13(e1091)* Munc13 (*pink*). **(E)** Average aldicarb time course for wild type (*black*), *cpx-1* null mutant (*red*), and two SNARE null mutants rescued with mutated SNARE domains. The *snb-1* synaptobrevin null mutant (*js104*) was rescued with mutations in three complexin-binding residues (3A, *blue*) as indicated in **(E)**. The *unc-64* syntaxin 1 null mutant (*js115*) was rescued with mutations in five complexin-binding residues (5A, *green*). Note that SNARE mutants lacking the ability to bind complexin phenocopy the *cpx-1* mutant. **(F)** Voltage-clamp recordings of spontaneous neuromuscular junction cholinergic fusion events in the absence of external calcium for wild type, *cpx-1* mutants, and rescue of *cpx-1* with a variant lacking its last 12 residues (Δ12) as indicated. Blue dots indicate individual vesicle fusion events. **(G)** Plot of percent rescue of spontaneous fusion rates versus percent rescue of aldicarb sensitivity (based on *t*_0.5_) for several distinct *cpx-1* mutants. This data was reanalyzed from Martin et al. (2011) (*red*), Wragg et al. (2013) (*green*), and Radoff et al. (2014) (*blue*) as indicated by symbol color. Data are mean ± SEM. ^∗∗^*p* < 0.01 by Tukey–Kramer test for multiple comparisons. *n.s.* = not significant.

**FIGURE 3:**
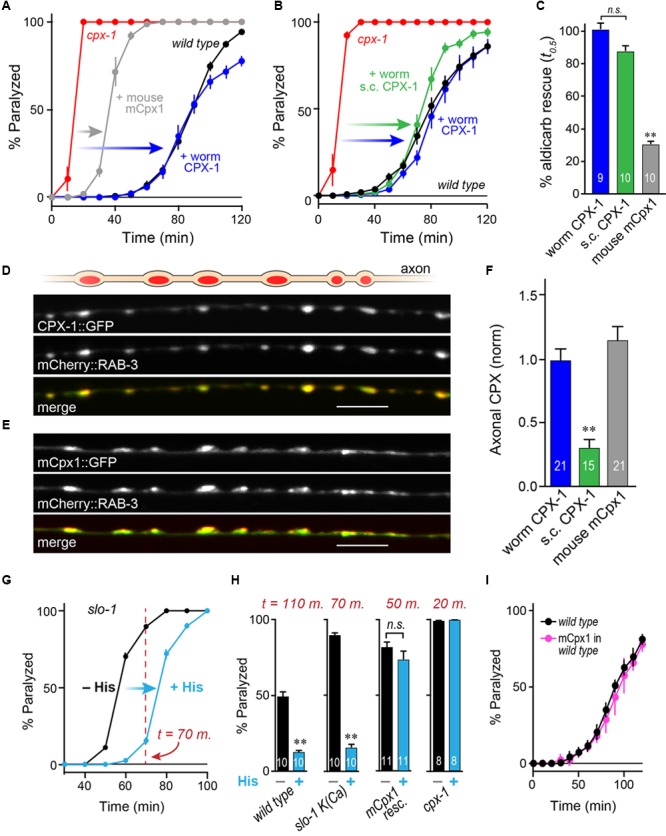
**Mouse complexin 1 fails to inhibit secretion in worm synapses. (A)** Aldicarb paralysis time course for wild type (*black*), *cpx-1* mutant (*red*), and *cpx-1* mutant rescued with either worm CPX-1 (*blue*) or mouse mCpx1 (*gray*). **(B)** Aldicarb paralysis comparison of wild type (*black*), *cpx-1* null mutant (*red*), and *cpx-1* rescue using either a multi-copy array integrant (CPX-1, *blue*) or a single-copy integrant (s.c. CPX-1, *green*). **(C)** Percent rescue based on 50% paralysis time point for a multi-copy array of worm CPX-1 (*blue*), multi-copy array of mouse Cpx1 (*gray*), and a single-copy array of worm CPX-1 (*green*). **(D)** Representative confocal images of dorsal cord axonal worm CPX-1:: GFP (*Top*) co-expressed with mCherry::RAB-3 (*Middle*), and a merged display of both images (*Bottom*). Scale bar is 5 μm. **(E)** Representative confocal images of axonal mouse Cpx1:: GFP (*Top*) co-expressed with mCherry::RAB-3 (*Middle*), and a merged display of both images (*Bottom*). **(F)** Quantification of axonal protein abundance for the multi-copy arrays of worm CPX-1 (CPX-1, *blue*) and mouse Cpx1 (Cpx1, *gray*) as well as the single-copy array of worm CPX-1 (s.c. CPX-1, *green*), normalized to an internal fluorescent standard (see Materials and Methods). **(G)** Aldicarb paralysis time course of *slo-1* K(Ca) mutants expressing the HisCl channel under a cholinergic promoter in the presence (*blue*) and absence (*black*) of histamine. **(H)** Summary of aldicarb paralysis in the absence and presence of histamine for three genetic backgrounds all expressing the HisCl channel: wild type (*Left*), *slo-1* K(Ca) mutant (*Middle*), and *cpx-1* expressing mCpx1 (*Right*). The particular time point in the aldicarb assay is indicated above the bars for each genotype. **(I)** Aldicarb paralysis time course for wild type (*black*) or mCpx1 over-expressed in wild type animals (*pink*). Data are mean ± SEM. ^∗∗^*p* < 0.01 by Tukey–Kramer test for multiple comparisons in **(C,F)** or Student’s *t*-test for **(H)**. *n.s.* = not significant.

We first examined highly homologous regions of complexin shared between mouse and worm. The major defining feature of complexin is its CH, the 25 residue alpha-helical region of complexin that directly binds to the assembled SNARE complex (**Figure 1A**). This is by far the most conserved domain of complexin and shows comparable conservation to the SNARE domains of the neuronal SNARE proteins – especially VAMP2 and Syntaxin 1, its two binding partners (**Figure 1B**). The SNARE domains of VAMP2 and Syntaxin 1 share 87 and 85% sequence identity, respectively, between *C. elegans* and mouse. Likewise, the CH of *C. elegans* CPX-1 is 76% identical to that of mouse complexin 1 (hereon referred to as mCpx1). The other protein domains of complexin are far less conserved as shown in **Figure 1C**. Several crystal structures of complexin bound to the ternary SNARE complex as well as biochemical and *in vivo* studies have identified specific residues required for the tight association of the CH and the ternary SNARE bundle, and these residues are almost perfectly conserved throughout phylogeny (Nonet et al., 1998; Saifee et al., 1998; Bracher et al., 2002; Chen et al., 2002; Giraudo et al., 2006; Xue et al., 2007; Maximov et al., 2009) (**Figure 1D**). To disrupt CPX-1 binding, several of these conserved SNARE residues were mutated to alanines (**Figure 1E**). We assessed the impact of perturbing CH binding at cholinergic synapses in *C. elegans* employing acute sensitivity to the cholinesterase inhibitor aldicarb by quantifying the rate of paralysis upon exposure to 1 mM aldicarb (**Figure 2A**). Numerous studies have established that impairment of ACh release decreases sensitivity to aldicarb and slows the rate of paralysis, whereas hypersecretory mutations accelerate paralysis (Rand and Russell, 1985; Miller et al., 1996; Nurrish et al., 1999; Mahoney et al., 2006; Martin et al., 2011). As described previously, worms rapidly paralyzed in the absence of complexin due to a high rate of spontaneous SV fusion (Hobson et al., 2011; Martin et al., 2011; Wragg et al., 2013). This hypersecretion was fully suppressed to wild-type levels by expressing a full-length CPX-1 in all neurons (**Figure 2B**). Both deletion of the central helix (ΔCH) and substitution of two key residues in the central helix (KY/AA) completely eliminated CPX-1 inhibitory function by this assay (**Figures 2B,C**). Note that all CPX-1 variants used in this study were tagged with a C-terminal GFP and that the full-length CPX-1:: GFP fusion protein fully rescued *cpx-1* mutants either as a multi-copy array or single-copy integrant (**Figure 3B**) (Martin et al., 2011; Wragg et al., 2013). Synaptic expression levels were assessed by imaging fluorescence in the dorsal nerve cord for all strains, and representative measurements of expression for several strains are shown in **Supplementary Table S1**. All strains analyzed in this study expressed complexin at higher levels than the single-copy integrant.

In principle, the hypersecretion observed in *cpx-1* mutants could emerge from an independent secretion pathway unrelated to canonical SV fusion at the synapse. Perhaps an unanticipated change in trafficking in the absence of CPX-1 could account for the hypersensitivity to cholinesterase inhibitors. However, the additional secretion events observed in *cpx-1* mutants relied on the same exocytosis machinery as in wild-type animals since SNARE hypomorphic mutants in *snb-1* synaptobrevin 1 and *unc-64* syntaxin 1 strongly suppressed the hypersecretion phenotype of *cpx-1* (**Figure 2D**). Furthermore, a weak hypomorphic mutant in the critical SV fusion protein *unc-13* Munc13 also suppressed *cpx-1* (**Figure 2D**). These observations indicate that neurotransmitter secretion remained highly sensitive to the neuronal SNAREs and essential SNARE-binding proteins in the absence of CPX-1.

The deep conservation of the SNARE residues that interact with the CH domain suggests that complexin inhibition relies on this interaction. To test for this possibility, we rescued neuronal SNARE mutants with mutated SNARE proteins designed to eliminate complexin binding (Maximov et al., 2009). Three complexin-binding residues of the VAMP2 ortholog SNB-1 were mutated to alanine (DLV/AAA = ‘3A’), and this construct was expressed in *snb-1* null mutants to replace endogenous vSNAREs. The SNB-1(3A) constructs fully phenocopied *cpx-1* null mutants in the presence of endogenous complexin (**Figures 1E, 2E**). Similar results were reported for mCpx1 in a previous study (Maximov et al., 2009). Likewise, a 5-residue mutant of the Syntaxin 1 ortholog UNC-64 (LMDMD/AAAAA = ‘5A’) expressed in the *unc-64* null mutant background identically phenocopied *cpx-1*. Both of these SNARE variants were functional since the transgenic animals expressing them were living, highly mobile, and displayed excessive ACh secretion, whereas null mutants in either *snb-1* or *unc-64* die at an early larval stage (Nonet et al., 1998; Saifee et al., 1998). Furthermore, hypomorphic alleles of these SNAREs are known to be severely uncoordinated and display strong resistance to aldicarb (Miller et al., 1996; Nonet et al., 1998; Saifee et al., 1998). Thus, the aldicarb hypersensitivity phenotype of *cpx-1* arose specifically from the loss of a complexin–SNARE interaction rather than through some unidentified complexin function. Prior electrophysiological studies in *cpx-1* mutants demonstrated that spontaneous fusion in the absence of external calcium is highly elevated when complexin function is impaired (**Figure 2F**) (Hobson et al., 2011; Martin et al., 2011; Wragg et al., 2013; Radoff et al., 2014). Replotting data from several of these studies against the percent rescue of spontaneous fusion rate versus the percent rescue of aldicarb sensitivity for a variety of CPX-1 structural mutations revealed a strong correlation (**Figure 2G**). The acute aldicarb sensitivity assay therefore provides a reasonable quantitative assessment of CPX-1 inhibitory function in the context of an intact behaving animal.

### Mouse Complexin 1 Fails to Inhibit Secretion in Worm Synapses

To examine the functional conservation of complexin across distantly related species, mouse mCpx1 was expressed as a multi-copy array in the nervous system of *C. elegans cpx-1* mutants lacking endogenous CPX-1. These transgenic animals exhibited only a small degree of functional rescue based on aldicarb sensitivity compared to either single-copy CPX-1:: GFP (s.c. CPX-1) or over-expressed CPX-1:: GFP (**Figures 3A–C**). Failure to rescue could have resulted from poor protein expression or non-synaptic localization of mouse complexin. However, mCpx1:: GFP synaptic localization was similar to CPX-1:: GFP when co-expressed with a SV marker (**Figures 3D,E**). Furthermore, mCpx1:: GFP synaptic abundance was quantitatively similar to CPX-1:: GFP (**Figure 3F**). The GFP fusion itself did not impair mCpx1 function because untagged mCpx1 also failed to rescue (data not shown). Thus, mCpx1 failed to restore proper function in *cpx-1* mutants, and neither expression levels nor mislocalization could account for this failure.

An important difference between mammalian and invertebrate complexins is the relative impact on promoting calcium-triggered release versus inhibiting spontaneous fusion. In mouse, loss of mCpx1/2 causes a significant decrease in calcium-triggered fusion while spontaneous fusion is either increased or decreased over a broad range depending on the neuronal subtype and perhaps on the methodologies employed (Xue et al., 2007; Maximov et al., 2009; Strenzke et al., 2009; Lin et al., 2013; Yang et al., 2013). Expression of mCpx1 in fly synapses significantly boosts calcium-triggered neurotransmitter release (Cho et al., 2010). However, in both worm and fly, the most conspicuous effect of losing complexin is a profound increase in the rate of spontaneous fusion (Huntwork and Littleton, 2007; Cho et al., 2010; Hobson et al., 2011; Martin et al., 2011; Wragg et al., 2013). Accordingly, the hypersecretion observed in worm *cpx-1* mutants expressing mCpx1 could arise from an upregulation of calcium-triggered release rather than a failure to suppress spontaneous release. To examine this possibility *in vivo*, we expressed a fly histamine-gated chloride channel (HisCl) in worm cholinergic neurons and performed aldicarb sensitivity assays in the presence and absence of histamine (Pokala et al., 2014). Partial silencing of cholinergic neurons by activation of hyperpolarizing HisCl channels would be expected to decrease calcium-triggered ACh release and to delay paralysis on aldicarb relative to control animals. To demonstrate this effect, HisCl was expressed in *slo-1* K(Ca) channel mutants (**Figure 3G**). These mutants are hypersensitive to aldicarb due to elevated calcium-triggered secretion in the absence of a repolarizing K(Ca) current (Wang et al., 2001; Martin et al., 2011). As anticipated, the addition of histamine significantly decreased ACh secretion in *slo-1* mutants as well as wild-type animals (**Figure 3H**). However, the same treatment had no effect on either *cpx-1* mutants or *cpx-1* mutants expressing mCpx1, suggesting that the enhanced secretion observed in these transgenic animals derives from enhanced spontaneous fusion rather than increased calcium-triggered fusion (**Figure 3H**). Finally, no enhancement of secretion was observed when mCpx1 was over-expressed in wild-type animals to determine if this variant could drive additional secretion via its facilitatory function (**Figure 3I**). Taken together, these experiments indicate that a conventional spontaneous SV fusion pathway is strongly elevated in *cpx-1* null mutants and that mCpx1 suppresses this fusion pathway to only a small extent despite being highly expressed and properly localized to worm synapses.

### The C-terminal Domain of Mouse Cpx1 Accounts for Its Failure to Inhibit in Worm

**FIGURE 4:**
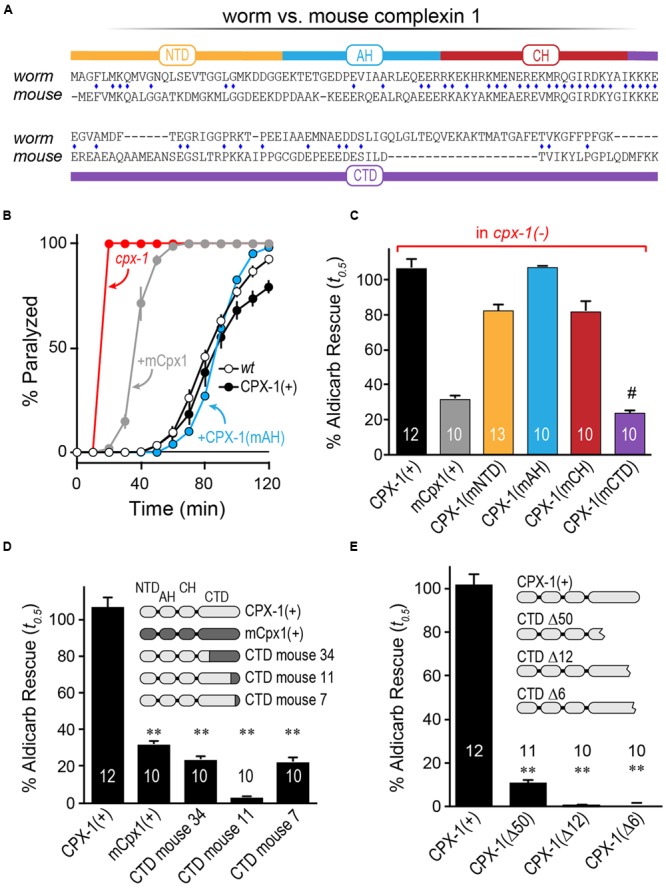
**The C-terminal domain of mouse Cpx1 accounts for its failure to inhibit in worm. (A)** Protein sequence alignment for worm CPX-1 and mouse Cpx1 showing the N-terminal domain (NTD), accessory helix domain (AH), central helix domain (CH), and C-terminal domain (CTD). Identical residues are indicated with a blue diamond. **(B)** Aldicarb time course for wild type (*black, open circles*), *cpx-1* (*red*), and *cpx-1* rescued with either worm CPX-1 (+CPX-1, *black, filled circles*), mouse Cpx1 (mCpx1, *gray*), or a chimeric worm CPX-1 variant containing the mouse accessory helix [+CPX-1(mAH), *blue*]. **(C)** Summary of aldicarb rescue of *cpx-1* mutants (by 50% paralysis time) for full-length worm CPX-1 (*black*), mouse Cpx1 (*gray*), and four chimeric worm CPX-1 variants containing mouse Cpx1 domains: mouse NTD (*orange*), mouse AH (*blue*), mouse CH (*red*), and mouse CTD (*purple*). **(D)** Summary of aldicarb rescue of *cpx-1* mutants by 50% paralysis time for full-length worm CPX-1 (*light gray*), mouse Cpx1 (*dark gray*), and three CTD chimeras containing various lengths of mouse Cpx1 substituted for the corresponding worm CPX-1 sequence as indicated (*light/dark gray*). **(E)** *cpx-1* mutant rescue with worm CPX-1 harboring various deletions in the CTD lacking the last 50 residues (Δ50), last 12 residues (Δ12), or last 6 residues (Δ6). Data are mean ± SEM. ^∗∗^*p* < 0.01 by Tukey–Kramer test for multiple comparisons and ^#^not significantly different from rescue with full=length mCpx1.

Having established that mCpx1 fails to inhibit SV fusion at worm synapses, we next explored individual complexin domains within mCpx1 to identify which domains failed to substitute for their homologous worm complexin domains (**Figure 4A**). Each domain of worm CPX-1 was substituted with the corresponding region of mCpx1 and expressed in *cpx-1* null mutants to assess the degree of functional rescue by aldicarb sensitivity. As shown in **Figure 4B**, some substitutions such as the AH domain fully restored wild-type complexin function (Radoff et al., 2014). In fact, of the four protein domains within complexin, only introduction of the mouse CTD recapitulated a failure to restore function to the same degree as full-length mCpx1 (**Figure 4C**). These findings indicate that the lack of functional rescue originates in the CTD of mCpx1. To further explore this region, several chimeras with varying lengths of the mCpx1 C-terminus were expressed in *cpx-1* null mutants. We found that even replacing only the last six residues of worm CPX-1 with the corresponding mouse mCpx1 residues strongly impaired the inhibitory function of CPX-1 (**Figure 4D**). If the majority of the CTD sequence was deleted rather than replaced, complexin function was impaired to a similar extent, suggesting that adding back mouse complexin residues failed to restore functionality lost with the deleted worm residues (**Figure 4E**). Thus, the highly divergent CTD of complexin in mouse and worm accounts for the lack of functional rescue.

### Monitoring Membrane Interactions with a C-terminal Tryptophan

**FIGURE 5:**
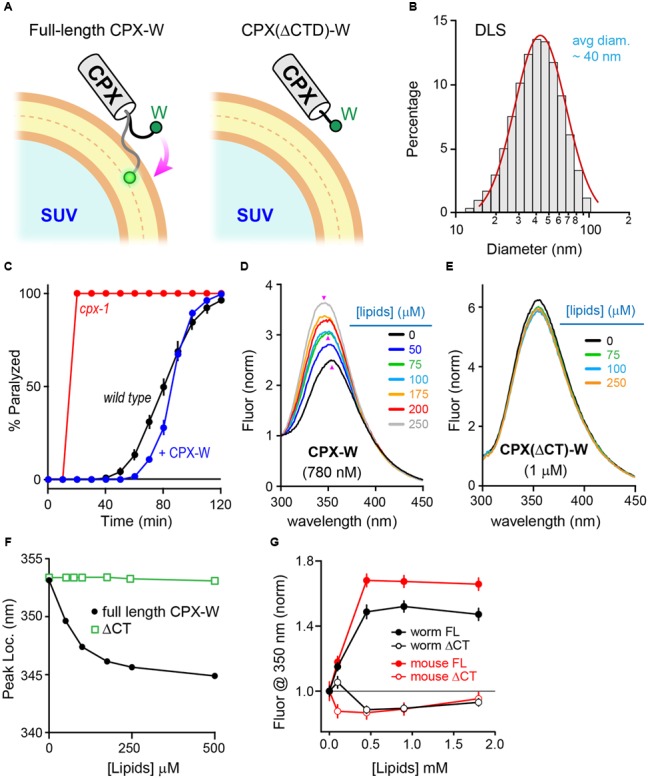
**Monitoring membrane interactions with a C-terminal tryptophan. (A)** Cartoon of the CPX-1 C-terminal region containing a tryptophan (W) binding a small unilamellar vesicle (SUV) either with (full-length) or without the last 50 residues of CPX-1 (ΔCTD). Note that tryptophan fluorescence at 350 nm increases when it penetrates into the lipid bilayer (White ref). **(B)** Representative dynamic light scattering (DLS) data for the SUVs used in this study. SUVs containing 85% POPC and 15% POPS were prepared by sonication (see Materials and Methods for details). The mean SUV diameter was estimated to be 35 ± 4 nanometers for this sample. Histogram was fit to a log-normal distribution (*red*). **(C)** Aldicarb time course for wild type (*black*), *cpx-1* mutants (*red*), and *cpx-1* mutants rescued with a CPX-1 harboring a C-terminal tryptophan (CPX-W, *blue*). **(D,E)** Emission spectrum for recombinant full-length worm CPX-W or CPX-W lacking the last 50 residues (ΔCT) excited at 280 nm in the presence of varying concentrations of lipids as indicated. Data is normalized to the fluorescence measured at 300 nm after background subtraction. Emission peaks are indicated with pink arrowheads. **(F)** Location of the emission peak is plotted as a function of lipid concentration for full-length CPX-W (*black*) and CPX-W lacking its C-terminal 50 residues (ΔCT, *green*). **(G)** Normalized emission fluorescence at 350 nm is plotted as a function of lipid concentration for worm CPX-1 (*black*) and mouse Cpx1 (*red*) for full-length constructs (*solid circles*) and variants lacking their C-terminal domains (*open circles*). Data are mean ± SEM.

Why does the CTD of mCpx1 fail to restore function in worm synapses? Previous studies in worm demonstrated that a major role of the CPX-1 CTD is to properly localize complexin to SVs via a membrane-binding region comprising the last ∼34 residues of CPX-1 (Wragg et al., 2013, 2015; Snead et al., 2014). mCpx1 also contains a membrane-binding region (Seiler et al., 2009; Snead et al., 2014; Gong et al., 2016), and this region is required for proper inhibition of spontaneous fusion in cultured mouse hippocampal neurons (Kaeser-Woo et al., 2012). The diameter of a typical SV is ∼30 nm in *C. elegans* and ∼40 nm in mammalian neurons, making it one of the most highly curved membranes within a cell (Rostaing et al., 2004; Takamori et al., 2006). To examine the membrane-binding properties of complexin on highly curved membranes *in vitro*, recombinant CPX-1 terminating with an added tryptophan (CPX-W) was incubated with small unilamellar vesicles (SUVs) and the fluorescence spectrum of tryptophan excited at 280 nm was monitored (**Figure 5A**). Typical SUV preparations comprised a relatively uniform population of vesicles with an average diameter of 35–45 nm as determined by dynamic light scattering (**Figure 5B**). Note that neither worm nor mouse complexin 1 contains endogenous tryptophan residues. CPX-1 preferentially binds to highly curved membranes irrespective of lipid head-group composition (Snead et al., 2014). The functionality of CPX-1 containing a C-terminal tryptophan was confirmed *in vivo* by rescue of *cpx-1* mutants with a CPX-W construct (**Figure 5C**). Thus, including a terminal tryptophan did not impair CPX-1 inhibition of ACh secretion. Full-length recombinant CPX-W incubated with increasing concentrations of SUVs exhibited a corresponding increase in peak emission fluorescence (**Figure 5D**). Furthermore, the location of the emission peak shifted toward shorter wavelengths at high lipid concentrations, consistent with tryptophan partitioning into the low dielectric environment of the SUV lipid bilayer (Ladokhin et al., 2000; Ladokhin and White, 2001). This membrane partitioning depended on the C-terminal region of CPX-1 as deletion of the last 34 residues (but retaining the C-terminal tryptophan) eliminated detectable increase in emission and peak blue-shift (**Figures 5E,F**), indicating that partitioning is dependent on and reflects membrane-binding by complexin. Corresponding *in vitro* experiments with mouse mCpx1-W confirmed that mammalian complexin also bound SUVs with a similar affinity in a manner strictly depending on the presence of the CTD (**Figure 5G**). Thus, the worm and mouse complexin 1 bound to SUVs with comparable affinities *in vitro* despite significant differences in their primary sequence.

### Impact of C-terminal Hydrophobic Residues

**FIGURE 6:**
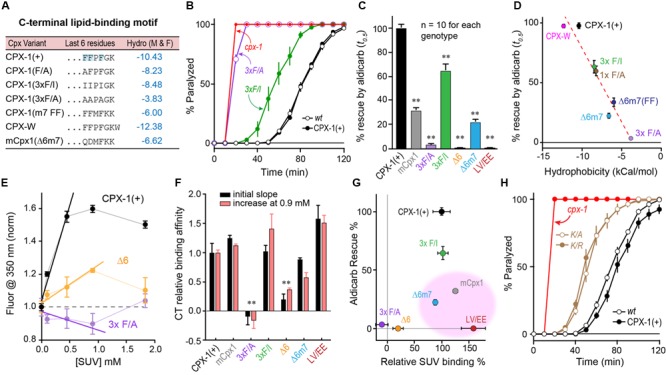
**Hydrophobic residues at the C-terminus are required for complexin function. (A)** The last 6–7 residues of several complexin variants are indicated along with a measure of their aggregate hydrophobicity using an empirical measure of side-chain ΔG moving from an aqueous to membrane lipid environment (Moon and Fleming, 2011) in units of kcal/mol. **(B)** Aldicarb time course for wild type (*black open circles*), *cpx-1* mutant (*red*), and *cpx-1* mutant rescued with either full-length worm CPX-1 (*black filled circles*), or with a CPX-1 variant substituting the terminal three phenylalanines with either alanine (3×F/A, *purple*) or isoleucine (3×F/I, *green*). **(C)** Summary of *cpx-1* mutant rescue using full-length worm CPX-1 (*black*), mouse Cpx1 (*gray*) or C-terminal variants: terminal three F to A (*purple*), F to I (*green*), deletion of the last 6 residues (Δ6, *orange*), substitution of the last 6 worm residues with the last 7 mouse Cpx1 residues (Δ6m7, *blue*), and an amphipathic domain double point mutation L117E V121E (LV/EE, *dark red*). **(D)** Plot of aldicarb rescue versus calculated hydrophobicity for the variants listed in **(A)** dashed line is linear fit with *r*^2^ = 0.93. **(E)** Summary of several tryptophan fluorescence measurements across a range of lipid concentrations for three representative CPX-W variants normalized to their peak emission at 350 nm in the absence of lipids. Full-length worm CPX-1 (*black*), CPX-1 lacking the last 6 residues (Δ6, *orange*), and CPX-1 with its terminal three phenylalanines replaced with alanines (3× F/A, *purple*). The initial slopes are measured by fitting the first 3 data points in each curve to a line. **(F)** Summary of relative membrane binding for the complexin variants in **(C)** as measured by either the initial slope (*black*) or by the fold increase in fluorescence at 0.9 mM lipids (*salmon*). **(G)** Percent aldicarb rescue is plotted versus percent relative lipid binding for the same complexin variants. The three variants with strong lipid binding but poor functional rescue are highlighted in pink. **(H)** Aldicarb time course for wild type (*black open circles*), *cpx-1* mutant (*red*), and *cpx-1* rescued with full-length worm CPX-1 (*black filled circles*) or CPX-1 with the terminal lysine replaced by either an alanine (K/A, *brown open circles*) or arginine (K/R, *brown filled circles*). Data are mean ± SEM. ^∗∗^*p* < 0.01 by Tukey–Kramer test for multiple comparisons.

Inspection of the primary amino acid sequence of the CTDs of worm and mouse complexin revealed little similarity beyond the previously described amphipathic region (L117 – K136 in worm CPX-1 and E114 – P125 in mouse mCpx1) (Wragg et al., 2013; Snead et al., 2014). To better understand the contribution of the last few residues to CPX-1 function and lipid binding, we explored several mutations, focusing on the three C-terminal phenylalanines characteristic of nematode complexins (**Figure 6A**). Structural studies (Snead et al., 2017) highlighted a potential role in membrane binding for this C-terminal motif. Several substitutions of one or more phenylalanines were made to alter the overall hydrophobicity of these last six residues, and the effective hydrophobicity was estimated using an empirical scale created by Moon and Fleming (2011). This scale was generated from measurements of the free energy change for moving an amino acid side chain from the cytoplasm to the middle of a lipid bilayer in the context of a folded transmembrane protein. All mutations in this C-terminal region of CPX-1 produced significant impairments in complexin inhibitory function as measured by aldicarb sensitivity (**Figures 6B,C**). Moreover, a strong correlation was observed between the hydrophobicity of the six residue C-terminal motif and the ability of CPX-1 to inhibit ACh secretion (**Figure 6D**). These same mutations were introduced into recombinant CPX-W to quantify the degree to which membrane binding was impaired. To monitor changes in binding affinity, we calculated both the initial slope of normalized fluorescence increase at 350 nm versus lipid concentration and the relative increase in fluorescence in the presence of 0.9 mM lipid versus lipid-free medium (**Figure 6E**). These approaches minimized inaccuracies encountered at high lipid concentrations due to light scattering (Ladokhin et al., 2000; Ladokhin and White, 2001). By either measure of binding affinity, the two perturbations that most strongly disrupted lipid binding were the deletion of the last six residues (Δ6) and the substitution of all three phenylalanines for alanines (3 × F/A) (**Figure 6F**). We noted that mCpx1 is considerably less hydrophobic than worm CPX-1 over the last six residues (**Figure 6A**), but surprisingly, replacing the last six CPX-1 residues with the last seven mCpx1 residues (Δ6m7) had only a modest impact on membrane binding. When the relative functionality of the C-terminal CPX-1 variants was plotted versus their relative membrane binding, three variants failed to show a correlation between membrane binding and CPX-1 inhibition (**Figure 6G** pink region). Full-length mCpx1, CPX-1 with the mCpx1 last seven residues, and a double point mutation in the amphipathic region (L117E V121E) all exhibited reasonably strong lipid binding but failed to rescue *in vivo*. Taken together with the other C-terminal mutations, these results demonstrate that membrane binding by the CTD is necessary but not sufficient for CPX-1 inhibitory function. Finally, since almost all known non-prenylated complexins terminate with a lysine, we substituted this lysine with either arginine (K/R) or alanine (K/A) and rescued *cpx-1* mutant animals with these variants (**Figure 6H**). Both substitutions significantly impaired CPX-1 inhibition, indicating that the conserved terminal lysine was required for CPX-1 inhibitory function. The *in vitro* and *in vivo* experiments described here demonstrate that although membrane binding by the CTD of complexin is critically important, other features of this region beyond membrane binding appear to play a role, and these features are poorly conserved between species.

### Molecular Dynamics of Membrane Interactions with the Complexin C-terminal Motif

**FIGURE 7:**
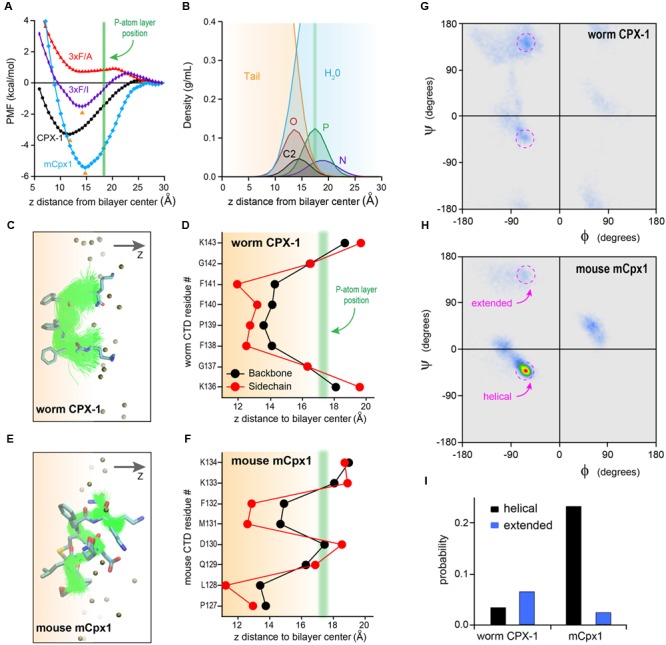
**Molecular dynamic simulations of complexin C-terminal peptides binding to the bilayer. (A)** Bilayer-binding PMFs of four peptides: CPX-1 = KGFPFFGK (*black*), 3×F/A = KGAPAAGK (*red*), 3×F/I = KGIPIIGK (*purple*), mCpx1 = PLQDMFKK (*blue*). The positions of free energy minima for CPX-1, 3×F/I, and mCpx1 are indicated with orange arrowheads and average phosphorous (P) atom position is indicated with a green vertical line. **(B)** Density profiles of lipid atoms in the head-group and linker regions and water molecules: N, nitrogen, P, phosphate, O, oxygen atoms of the glycerol group, C2 is the start of the two acyl chains, tail, all atoms of the two lipid tails. *X*-axis represents the vertical distance (z) away from the bilayer mid-plane along the bilayer normal. The vertical green line (same as in **A**) indicates the average z position of the P atoms. **(C,E)** Backbone conformation of bilayer-bound peptides. Atom color scheme: C (*cyan*), N (*blue*), O (*red*), S (*yellow*). Alignment of peptide backbone ensemble onto one representative conformation. **(D,F)** Average residue insertion depth for the backbone was measured as the average vertical distance of the Cα atom to the bilayer center, and the insertion depth for the sidechain was measured as the average vertical distance of the sidechain heavy atoms to the bilayer center. All snapshots were rendered using VMD (Humphrey et al., 1996). Ramachandran plot heat maps are shown for the worm CPX-1 peptide **(G)** and mCpx1 peptide **(H)** using the same color scale. The torsion angle regions outlined in magenta correspond to either helical or extended conformations as indicated. **(I)** The fractional occupancy of helical (*black*) or extended (*blue*) states was computed based on the proportion of torsion angles located in a 20° × 20° region centered on (–62°,–43°) for a right-handed helix or (–55°, +150°) for a beta strand.

Perhaps the simplest hypothesis for the failure of the mouse C-terminal motif to function when swapped into worm complexin is a large difference in hydrophobicity of these residues. However, *in vitro* membrane-binding experiments (**Figures 5, 6**) indicated that both the mCpx1 CTD and the Δ6m7 variant bound to membranes with a similar affinity to CPX-1 despite being significantly less hydrophobic. To further investigate the nature of complexin membrane interactions in a structural context, we performed all-atom MD simulations of peptides comprising the last eight residues of either worm CPX-1 or mouse mCpx1 in proximity to a lipid bilayer containing phosphatidylcholine (POPC), phosphatidylethanolamine (POPE), and phosphatidylserine (POPS) in a ratio of 60:25:15 (see Materials and Methods for details of the MD simulations). The simulations revealed that both worm and mouse Cpx peptides partitioned into the lipid bilayer and exhibited energetics that favored membrane binding to a similar degree. In fact, the mouse peptide displayed a somewhat deeper free energy trough than the worm peptide (-3.3 kcal/mol vs. -5.4 kcal/mol for worm and mouse, respectively) (**Figure 7A**). The energy minimum occurred at a penetration depth of 10 Å from the bilayer center for CPX-1 and 15 Å for mCpx1. The C-terminal motifs of both complexins entered the hydrophobic core of the lipid bilayer in accordance with the density profiles of the lipid head group atoms and water molecules as shown in **Figure 7B**. The structural organization of the peptides embedded in the lipid bilayer are indicated by the ensembles of peptide backbone conformations observed in the simulations for CPX-1 (**Figure 7C**) and mCpx1 (**Figure 7E**). To clarify the position of the side chains, the conformational ensemble cluster for each peptide is superimposed on a cluster representative for each complexin in the corresponding figure (**Figures 7C,E**). The typical configurations adopted by the two complexin peptides were strikingly different. Worm CPX-1 dipped uniformly into the hydrophobic core via its phenylalanines anchored by lysines snorkeling back to the head group layer on either side (**Figure 7D**). In contrast, mCpx1 adopted a helical bend with L128 and M131 directed into the bilayer while Q129 and D130 were directed toward the aqueous phase (**Figure 7F**). This difference in configuration was also quantified by computing the distribution of peptide backbone dihedral angles for CPX-1 and mCpx1 as shown in **Figures 7G–I**. Notably, a point mutation in L128 (L128M) of the human mCpx1 ortholog has been identified in a patient with significant intellectual disability, severe seizures, myotonia, and conductive hearing loss (Redler et al., 2017). In summary, MD simulations suggest that the final eight residues of mammalian complexin adopt a more structured configuration that promotes membrane binding despite the relatively low hydrophobicity of this region compared to nematode complexin.

The dissimilar bound states of the worm and mouse C-terminal motifs can stabilize distinct orientations and positions relative to the upstream peptide as well as alter the availability of the C-terminal side chains for other protein interactions. Moreover, there may be functionally significant kinetic differences in the binding and unbinding of worm and mouse complexin (see Discussion). Such a kinetic difference is made more likely given the critical role played by the three phenylalanines of worm CPX-1. While the free energy of binding is clearly dependent on their special mode of insertion (substitution of the phenylalanines with isoleucines diminished the estimated free energy of binding by twofold, whereas alanine substitutions eliminated the free energy trough altogether – **Figure 7A**), it is possible that their coordinated withdrawal from the lipid membrane could slow the unbinding process.

### NMR Spectroscopic Analysis of the C-terminal Domain

**FIGURE 8:**
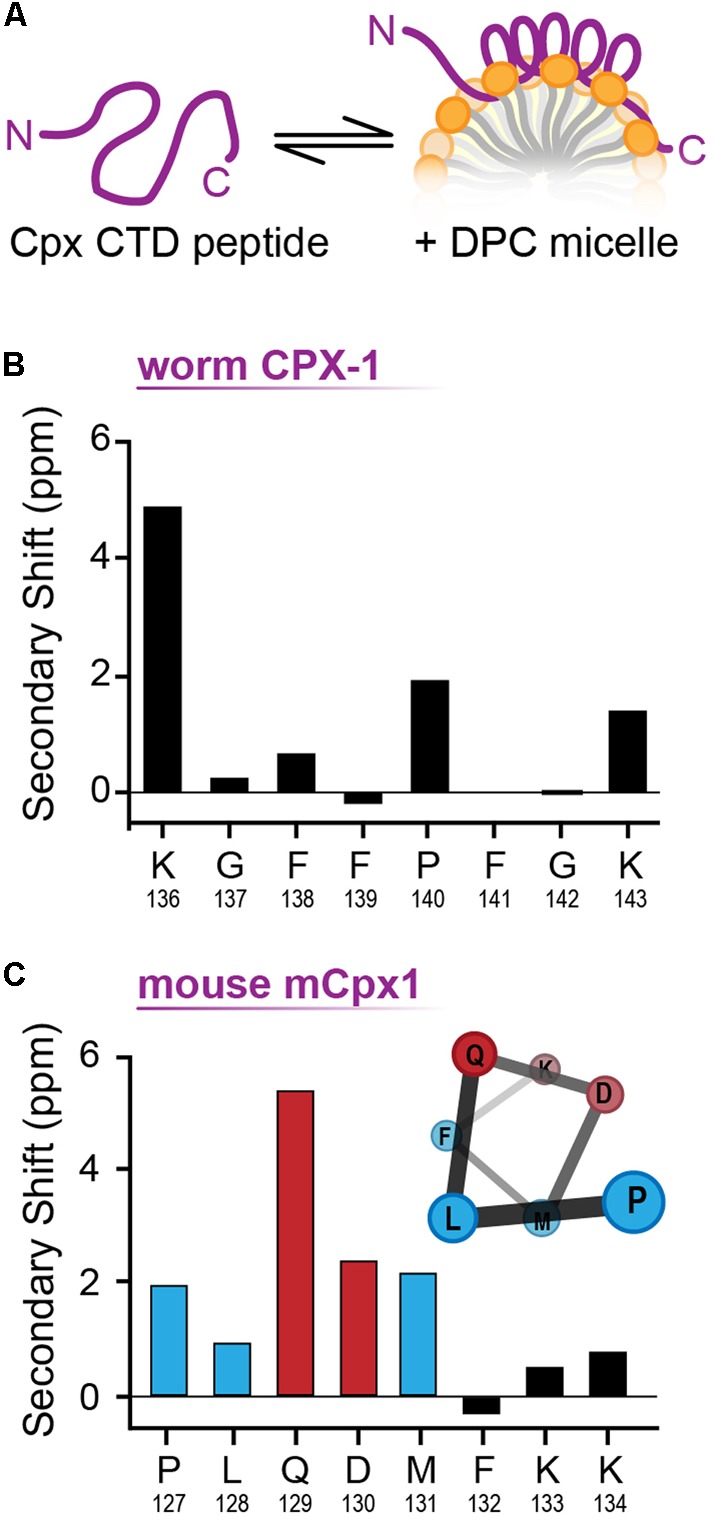
**NMR analysis of membrane bound CT motif structure. (A)** Cartoon of the complexin CTD peptide in solution (*Left*) and bound to a lipid micelle (*Right*). **(B)** Cα-Cβ shifts for the last nine residues of worm CPX-1 CTD peptide in the presence of 55 mM DPC. **(C)** Cα-Cβ shifts for the last eight residues of mouse mCpx1 CTD peptide in the presence of 55 mM DPC. Note the consecutive residues (blue and red bars) that display a positive chemical shift consistent with alpha helix formation as illustrated (*inset*). Hydrophobic residues are indicated in blue while polar/charged residues are depicted in red. The CTD peptide consisted of the last 53 residues for CPX-1 and 64 residues for mCpx1.

The structural differences between the C-terminal motifs of worm and mouse complexin in a membrane-like environment were further investigated with NMR spectroscopy. Worm CPX-1 and mCpx1 were incubated with dodecylphosphocholine (DPC) micelles (**Figure 8A**), a membrane mimetic that is amenable to solution-state NMR spectroscopy, and sequence-specific NMR backbone resonance assignments for both micelle-bound C-terminal motifs were obtained (Snead et al., 2017). Carbon chemical shifts for each C-terminal motif were then used to assess the degree of secondary structure in the micelle-bound state by calculating their deviation (secondary shift) from tabulated shifts characteristic of random coil behavior. In particular, positive carbon secondary shifts indicate alpha-helical structure (Wishart and Sykes, 1994). The six residues in the C-terminal motif of worm CPX-1 bound to DPC micelles in a configuration lacking any regular secondary structure, exhibiting small secondary shifts with no contiguous secondary shift patterns that might have suggested a transient helical structure (**Figure 8B**). In contrast, the C-terminal residues of mCpx1 exhibited a contiguous stretch of five positive carbon secondary shifts beginning with Proline 127 (**Figure 8C**), suggesting a significant population of helical structure in the mouse C-terminal motif. Thus, the NMR data corroborate the MD simulations, supporting the conclusion that a segment of the mCpx1 C-terminal motif interacted with lipids in the form of an alpha helical turn. The MD and NMR spectroscopy results, together with the results from mutagenesis studies (**Figures 4**–**6**), reveal significant differences between the C-termini of two Cpx1 orthologs when bound to membrane, and these differences profoundly affect complexin function. However, as the C-terminal motif is not the only region of the CTD exhibiting high variability across phylogeny, other regions may also account for functional differences between complexin isoforms, as discussed below.

### Conserved and Divergent Features of the Complexin C-terminal Amphipathic Region

**FIGURE 9:**
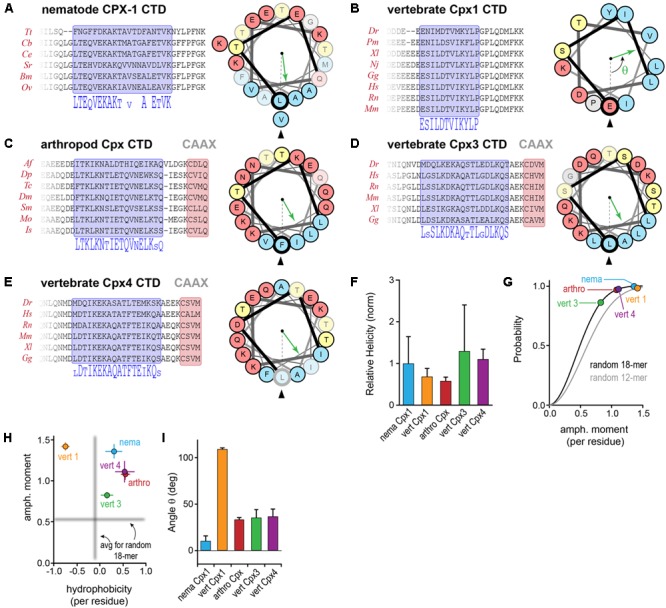
**Conserved and divergent features of the complexin C-terminal amphipathic region. (A)** Protein sequence alignments of nematode CPX-1 homologs across several nematode clades. A 20-residue amphipathic region is highlighted in blue, and the corresponding consensus sequence is shown below. A helical wheel representation of the amphipathic region depicts the spatial segregation of hydrophobic (*blue*) and hydrophilic (*yellow* and *red*) residues. The hydrophobic moment direction is indicated for *C. elegans* (*green arrow*) relative to the first residue in the amphipathic region (bold Leu117, *arrowhead*). See Section “Materials and Methods” for determination of the hydrophobic moment vector. Residues in the wheel that are poorly conserved within the phylum are faded. **(B–E)** Protein sequence alignments for several vertebrate Cpx1 homologs, arthropod Cpx homologs, and vertebrate Cpx3 and Cpx4 homologs together with helical wheel representations and amphipathic moments for representatives of each group (mouse Cpx1, *D. melanogaster* Cpx1, mouse Cpx3 and Cpx4). **(F)** The predicted helical tendency of the isolated amphipathic region was computed for each group using Agadir and normalized to the average nematode value. **(G)** Cumulative probability distributions of the amphipathic moment magnitude generated from one million random 18-mer peptide sequences (black line) and 12-mer peptide sequences (gray line) with the constraint that prolines were not allowed in the middle 14 residues to maintain a stable helical configuration. The phylogenetic average magnitude of the amphipathic moment vector divided by the number of residues in the region is shown for each of the four groups plotted on the distributions. The amphipathic moments for nematodes (*nema, blue*), arthropods (*arthro, red*), vertebrate Cpx1 homologs (*vert 1, orange*), vertebrate Cpx3 homologs (*vert 3, green*), and vertebrate Cpx4 homologs (*vert 4, purple*) occur in the 85–99th percentile, indicating relatively strong hydrophobic moments. **(H)** The amphipathic moment is plotted versus the overall hydrophobicity for each phylogenetic group as indicated. The mean amphipathic moment and hydrophobicity of random 18-mer peptides are shown in gray. **(I)** The average angle of the hydrophobic moment vector relative to the first residue in the amphipathic region is shown for each of the four groups. Error bars in **(F–I)** are standard deviation. See Supplementary Table S2 for a list of all species used in this analysis.

In addition to the C-terminal motifs explored above, upstream amphipathic regions of both worm CPX-1 and mCpx1 are known to adopt a helical conformation upon membrane binding and to confer selective binding to highly curved membranes such as SV membranes (Snead et al., 2014, 2017, co-submitted; Gong et al., 2016). In this region of the CTD, there is little or no primary sequence similarity between these two complexin orthologs. A systematic assessment of sequence and secondary structure conservation suggested that the CTD is in fact conserved within a particular phylum, but is highly divergent between phyla. For example, nematode CPX-1 and vertebrate mCpx1 homologs reveal a strong degree of intra-phylum conservation both at the primary sequence level and in the nature of the amphipathic sequence pattern (**Figures 9A,B**). To generate quantitative comparisons of both the strength and orientation of the amphipathic region, the amphipathic moment μ_H_ of each sequence, modeled as an alpha helix, was computed by vector addition from the moments of individual residues (each rotated 100° relative to the preceding residue) weighted by the hydrophobicity of the side chain (using the Moon & Fleming metric for hydrophobicity – see Materials and Methods), and normalized to the number of residues (Eisenberg et al., 1982). By definition, the amphipathic moment points toward the hydrophobic interaction surface (e.g., lipid bilayer) and its magnitude provides a measure of the degree of asymmetry between the hydrophilic and hydrophobic sides of the helix. The CAAX-box containing complexins harbored a conserved amphipathic region with a similar μ_H_ to other complexins (**Figures 9C–E**). Overall, the predicted helical tendency of the amphipathic region did not differ extensively between the representative phyla (**Figure 9F**). Compared to random peptides of identical length, the complexin amphipathic moment resided in the 85th99th percentile for all species analyzed (**Figure 9G**). Interestingly, the invertebrate complexins and the CAAX-box isoforms of the vertebrate complexins generally shared the same pattern of amphipathic residues and no net hydrophobicity (**Figure 9H**). In contrast, vertebrate Cpx1 (and Cpx2) exhibited both a higher amphipathic moment and a greater hydrophobicity distributed over a shorter stretch of residues (∼3 helical turns for vertebrate Cpx1/2 versus more than 5 turns for the other Cpx homologs). Moreover, the spatial orientation of the amphipathic moment of complexin 1/2 was markedly rotated when compared to the other homologs (**Figure 9I**). By these metrics, the invertebrate complexins and vertebrate Cpx3/4 isoforms were more similar to each other than to Cpx1/2. The broad conservation of the CTD amphipathic motif together with its functional importance in worm synapses suggests that this region generally plays an important role in proper complexin function. Is this function mechanistically conserved across phylogeny despite the differences described above?

### Substitutions and Rotations in the Amphipathic Region

**FIGURE 10:**
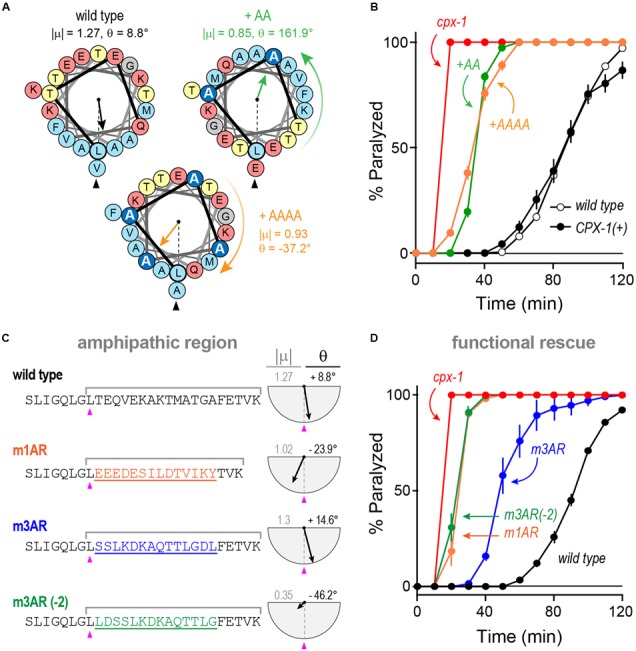
**Substitutions and rotations in the amphipathic region. (A)** Helical wheel depictions of the 20-mer amphipathic regions in wild-type worm CPX-1 as well as variants with either two alanines (+AA, *green*) or four alanines (+AAAA, *orange*) inserted at the start of the region just after the initial Leu 117 (*arrowhead*). The additional alanines are in dark blue. The amphipathic moment magnitude (| μ|) and angle (𝜃) are given for a 20-residue window beginning with L117 in the three variants. Note that two alanines invert the orientation of the amphipathic moment whereas four alanines approximately restore it. **(B)** Aldicarb time course for wild type (*black open circles*), *cpx-1* mutant (*red*), and *cpx-1* mutant rescued with full-length worm CPX-1 (*black filled circles*), or CPX-1 containing either two additional alanines (+AA, *green*) or four additional alanines (+AAAA, *orange*) following L117. **(C)** Chimeric amphipathic region substitutions were made in worm CPX-1 (*black*) using 14 residues from either mCpx1 (m1AR, *orange*), mCpx3 (m3AR, *blue*) or mCpx3 shifted two residues toward its N-terminus (m3AR –2, *green*) to perturb its amphipathic moment. For each sequence, the amphipathic moment was computed between L117 and the last lysine as indicated. The angle is measured relative to L117 as above. **(D)** Aldicarb time course for wild type (*black open circles*), *cpx-1* mutant (*red*), and *cpx-1* mutant rescued with CPX-1 containing either the mCpx1 amphipathic region (m1AR, *orange*), mCpx3 amphipathic region (m3AR, *blue*), or mCpx3 shifted by two residues (m3AR –2, *green*) as described in **(C)**. Data are mean ± SEM.

We explored the functional importance of the amphipathic region in worm CPX-1 first by introducing small perturbations to its structure and orientation as shown in **Figure 10A**. Either two or four consecutive alanines (+AA and +AAAA, respectively) were inserted just after the initial leucine of the amphipathic region (L117). The +AA insertion significantly rotated and diminished the μ_H_ whereas the +AAAA insertion produced a more subtle change in μ_H_. Despite their different effects on the amphipathic moment, both CPX-1 insertions equally failed to restore inhibitory function in the *cpx-1* null mutant (**Figure 10B**). Thus, the amphipathic region was highly sensitive to alterations in the primary amino acid sequence. Furthermore, chimeric substitutions between worm and mCpx1 failed to restore complexin function despite attempts to match the CPX-1 amphipathic moment (**Figures 10C,D**). Interestingly, the mouse mCpx3 amphipathic region restored about 50% of CPX-1 functionality. This rescue was highly sensitive to the precise mCpx3 sequence as a two-residue shift in the substituted region destroyed both the amphipathic moment and the functionality of this chimeric variant even though the amino acid content and most of the sequence were identical in these two chimeras (**Figures 10C,D**). Thus, even relatively minor alterations in the amphipathic region abolished CPX-1 inhibition whereas similar amphipathic regions from highly divergent complexin isoforms were able to restore the CPX-1 inhibitory function, but only when precisely substituted in the correct orientation.

## Discussion

It has proven difficult to deduce the structure/function relationship for complexin given the observed mixture of almost perfectly conserved regions and highly divergent domains. In the experiments described here, several features of complexin conservation were explored in the context of *in vivo* synaptic function using a simple behavioral assay as well as *in vitro* membrane binding assays. Five conclusions arise from these experiments. First, mouse complexin 1 fails to restore inhibitory function in worm synapses, and this is largely due to differences in the CTDs of mouse and worm complexin. Second, membrane-binding by several C-terminal residues is necessary but not sufficient for proper worm CPX-1 function. Third, the C-terminal motifs of worm and mouse complexins adopt distinct configurations, indicated by both MD simulations and NMR chemical shifts, perhaps explaining some of the failure of mCpx1 to function in worm CPX-1. Fourth, a deeply conserved amphipathic region is shared across both prenylated and non-prenylated complexins, with the vertebrate Cpx1/2 isoforms deviating from an otherwise characteristic pattern. Fifth, relatively subtle alterations in the amphipathic region can profoundly impact worm CPX-1 function, supporting the notion that the amphipathic region confers more than a generic membrane-binding capacity to complexin function. These results suggest that, in addition to membrane binding, there is another aspect of the complexin C-terminal region that has diverged across species.

### Conserved versus Divergent Regions of Complexin

Despite its small size, complexin displays a prominent heterogeneity in protein sequence conservation. The 25 residues comprising the CH are almost perfectly preserved both within phyla and between phyla. In effect, these residues define the complexin genes since the rest of the protein sequence has markedly diverged between phyla. To date, the only known binding partner of the CH is at the interface formed by the assembled synaptobrevin and syntaxin 1 SNARE helices in the ternary SNARE complex (Pabst et al., 2000; Bracher et al., 2002; Chen et al., 2002). Thus, the CH sequence is highly constrained by the equally conserved SNARE sequence (Kloepper et al., 2007). Examining secondary structure rather than primary sequence, conservation is more evident throughout the complexin protein. A stable alpha helix characterizes the AH domain across phylogeny (Radoff et al., 2014), while the lipid-binding CTD displays highly charged stretches, amphipathic helices, and hydrophobic regions in stereotyped locations (Snead et al., 2014, 2017, co-submitted). The AH conservation is both structural and functional since the mouse domain was fully operational in worm synapses when substituted into worm CPX-1 (Radoff et al., 2014). For the CTD, functional conservation is less clear. A previous study tested the impact of swapping mouse Cpx1 and fly Cpx (DmCpx) domains including the CTD (Xue et al., 2009). Introducing the fly C-terminus onto mCpx1 enhanced mCpx1 suppression of spontaneous neurotransmitter release in hippocampal autaptic cultures, consistent with the notion that invertebrate CTDs endow Cpx with a potent inhibitory function (Xue et al., 2009). Of note, five cases of homozygous mutations in the human Cpx1 ortholog *CPLX1* have been reported, and all involve either truncations that effective delete the CTD or a point mutation near the end of the CTD (Karaca et al., 2015; Redler et al., 2017). These mutations are associated with severe epilepsy as well as intellectual disability.

Across the animal kingdom, the Cpx superfamily can be divided into prenylated versus non-prenylated Cpx isoforms (Reim et al., 2005, 2009; Brose, 2008; Yang et al., 2015). While the *Drosophila* genome harbors only a single complexin gene, alternative splicing produces both prenylated and non-prenylated DmCpx variants (Buhl et al., 2013; Cho et al., 2015). The sequence analysis and structural comparisons presented here suggest that both prenylated and non-prenylated complexins share attributes in the CTD across phylogeny with the most divergence arising from vertebrate Cpx1/2 isoforms. Of particular interest, mammalian and fish Cpx3/4 isoforms, which are prenylated and limited in expression to specialized nervous tissue such as the retina, show functional similarity to the invertebrate complexins in their suppression of neurotransmitter release (Vaithianathan et al., 2013, 2015; Mortensen et al., 2016). Interestingly, a recent study utilized a distantly related cnidarian Cpx isoform from *Nematostella vectensis* (NvCpx1) in mammalian synapses and concluded that this non-bilaterian lineage Cpx lacked inhibitory activity while still facilitating calcium-triggered fusion (Yang et al., 2015). The Cpx CTDs of *Nematostella* and other basal animals (such as *Trichoplax*) lack an obvious amphipathic helix motif, suggesting that this aspect of CTD function might not be conserved outside of bilateria. The experiments presented here are consistent with these observations and indicate a correlation between Cpx inhibitory function and amphipathic properties of the CTD. We propose that the ancestral bilaterian Cpx homolog mediated an inhibitory function at the synapse via its CTD and that this function was attenuated in chordates for Cpx1/2 homologs. As these complexins subserve the central and peripheral nervous systems in vertebrates, loss of a constitutive inhibitory function in Cpx may have coincided with other specializations of the vertebrate nervous system relevant to the physiology of vertebrate chemical synapses. This raises the question of whether the inhibitory role of Cpx has been eliminated entirely in vertebrate Cpx1/2 isoforms. The inhibitory role of Cpx has not been entirely lost in the vertebrate central nervous system since inhibitory function of mammalian Cpx1/2 isoforms has been observed in hippocampal neurons and at the calyx of Held (Maximov et al., 2009; Kaeser-Woo et al., 2012; Chang et al., 2015). In addition, expression of mCpx1 in fly synapses lacking endogenous DmCpx rescued Cpx inhibitory activity to a large degree, indicating that mCpx1 retains some inhibitory activity that can operate in a distantly related synapse (Cho et al., 2010). Regardless of the evolutionary pressures that reshaped Cpx1/2 function in vertebrates, it is clear that alterations in C-terminal structure and function have a profound impact on the regulation of synaptic transmission, and a better understanding of this mechanism will shed light on the molecular control of SV fusion at all synapses.

### Membrane-Binding Properties of Complexins

Previous studies have established that both nematode and mammalian Cpx1 isoforms bind to either flat or curved membranes, but with a preference for relatively high curvature. This curvature-sensitive binding is accompanied by a transition of the C-terminal amphipathic region from an unstructured configuration to an alpha helix, with some similarity to a protein motif known as an ALPS domain (Antonny, 2011; Snead et al., 2014). Moreover, this conformational switch from disordered to helical is required for efficient CPX-1 inhibitory function in worm synapses (Snead et al., 2014). The last few residues of nematode complexin contain a hydrophobic stretch that binds to membranes irrespective of curvature, and this interaction is also required for CPX-1 inhibition. In contrast, the corresponding residues in mCpx1 lack the same degree of hydrophobicity but as shown here, appear to adopt an amphipathic helical turn that mediates comparable membrane binding. Interestingly, substituting the mCpx1 motif into worm CPX-1 lacking its final hydrophobic stretch of residues failed to restore CPX-1 inhibitory function *in vivo* even though this chimeric protein bound to SUVs with a similar affinity to CPX-1 as measured by tryptophan fluorescence. Based on our findings described here, there are several possible explanations for this failure to function.

First, although equilibrium binding does not appreciably differ between these two complexin isoforms, the binding kinetics may differ significantly. Little is currently known about the details of complexin binding kinetics (Gong et al., 2016), and yet the rate at which complexin binds or unbinds vesicle membrane may have functional consequences at the synapse. Indeed, a major inhibitory effect of mammalian complexin appears to be limited to a small time window during the priming process (Chang et al., 2015).

Second, the tryptophan fluorescence measurements only probe membrane interactions in pure lipid membranes whereas complexin interactions *in vivo* involve SVs packed with both integral membrane proteins and membrane-associated proteins (Takamori et al., 2006). It is possible that divergence of other protein interactions required for *in vivo* function accounts for the failure of mammalian Cpx C-terminal motif to replace the nematode motif *in vivo*. For instance, the mCpx1 C-terminus presented an electrostatic interaction surface mediated by Q129 and D130 directed out of the lipid bilayer in MD simulations that was not observed with the worm CPX-1 C-terminus, possibly capable of binding other synaptic proteins.

Third, the C-terminal tryptophan only reports the local membrane-binding behavior of the peptide. Other aspects of the membrane interaction within the amphipathic region or even the NTD may differ in this chimera without altering the C-terminal tryptophan fluorescence (Lai et al., 2016). The MD simulations suggested that the relative position of the C-terminal motif compared to the amphipathic region may differ significantly between worm and mouse complexin. In particular, side chains located six and seven residues upstream of the C-terminus (K136 G137) resided close to the membrane surface, whereas the corresponding residues of the mouse CT motif (P127 L128) were buried nearly 10 angstroms into the membrane. This difference in membrane penetration may alter the position of the neighboring amphipathic helix. However, since even when the entire CTD region was swapped between mouse and worm, the mouse version failed to rescue synaptic inhibition, a disruption of the coupling between the amphipathic region and CT motif cannot fully explain the species differences.

Finally, C-terminal prenylation is a common feature of many complexin isoforms, and its biological role is currently not well understood beyond a synaptic targeting function (Reim et al., 2005; Buhl et al., 2013; Iyer et al., 2013). Disruption of prenylation impairs Cpx inhibitory function, but it is still unclear what membrane-binding characteristics are endowed by prenylation in any complexin isoform (Reim et al., 2005, 2009; Cho et al., 2010; Buhl et al., 2013; Iyer et al., 2013).

### Model of Complexin Inhibitory Action at the Synapse

A number of previous studies have contributed to the growing knowledge of complexin membrane interactions and their role in complexin function (Seiler et al., 2009; Kaeser-Woo et al., 2012; Wragg et al., 2013, 2015; Snead et al., 2014; Gong et al., 2016).

**FIGURE 11:**
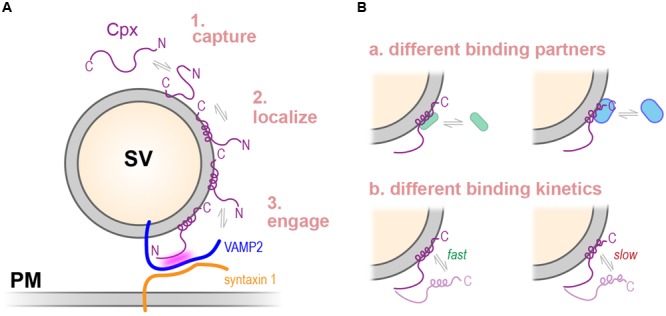
**Hypothetical sequence of events in complexin-mediated inhibition of vesicle fusion. (A)** Complexin is transiently captured via synaptic vesicle (SV) membrane interactions with the last few residues of its C-terminal domain (1). On highly curved vesicles such as small synaptic vesicles, the amphipathic region adopts an organized alpha-helical structure, perhaps as part of a mechanism for stabilizing other protein interactions and properly localizing complexin (2). The central helix of complexin binds the assembling SNARE bundle to prevent full assembly and spontaneous fusion (3). **(B)** Complexins from divergent species possess similar lipid- and SNARE-binding properties but other interactions depending on the C-terminal domain may not be well conserved. Potential differences in C-terminal binding partners (*a*) or kinetics of C-terminal membrane binding (*b*) may account for functional differences across phylogeny. SV, synaptic vesicle; PM, plasma membrane.

We previously showed that two membrane-binding modules within the CTD of complexin direct complexin to SVs. Moreover, upon binding to highly curved membranes, the amphipathic region adopts a functionally important alpha-helical structure (Snead et al., 2014). We propose that this helical conformation interacts with other proteins that help complexin to engage the assembling SNARE complex as vesicles dock and prime (**Figure 11A**). Based on observations described here and in other studies, we conclude that there are structural differences between the amphipathic and C-terminal motifs of worm CPX-1 and mouse mCpx1 (Snead et al., 2014, 2017). We further speculate that these differences are associated with non-conserved protein–protein interactions, so that the differences in conformational and sequence requirements for these interactions explain the failure of mCpx1 to restore proper inhibitory function in worm synapses (**Figure 11Ba**). Another possibility is that differences in the kinetics of membrane binding and unbinding by the CTD that result from the conformational and sequence differences may lead to the observed functional consequences (**Figure 11Bb**). A recent study revealed a potent but transient inhibitory function for mCpx1 during vesicle priming (Chang et al., 2015). It is possible that, in vertebrate synapses, complexin inhibition is required only during the priming process, whereas a longer term association of complexin and the fusion machinery is utilized in invertebrate synapses to minimize spontaneous fusion. The CTD provides a means of controlling the inhibitory strength of complexin, and the phylogenetic diversity of C-terminal sequences may reflect evolutionary divergence of synaptic regulation. It is noteworthy that in addition to its inhibitory activity, complexin has a separate positive role in calcium-triggered fusion. This function appears to be universally shared among all complexin isoforms, although a detailed understanding of this facilitatory mechanism is currently lacking (Xue et al., 2009; Cho et al., 2010; Martin et al., 2011; Yang et al., 2015; Trimbuch and Rosenmund, 2016).

### Controversies Regarding Complexin Function

Beginning with the first reports of complexin inhibitory activity in invertebrates, a stark contrast has persisted between mammalian and invertebrate complexin function at the synapse. Studies in numerous model synapses over the past decade have produced some consensus on the positive role of complexin in calcium-triggered neurotransmitter release as well as on the absolute dependence of this function on the SNARE-binding CH domain (Xue et al., 2007; Cho et al., 2010; Martin et al., 2011; Trimbuch and Rosenmund, 2016). However, the inhibitory function of complexin varies across synapses and across species, perhaps reflecting diverse demands on synaptic function in these different contexts. The work presented here emphasizes the contribution of two membrane-binding modules within the CTD of complexin as major drivers of this functional diversity. Nevertheless, the mechanistic basis of both the facilitatory and inhibitory roles of complexin remains poorly understood. Future studies will be required to place the diverse functions of the CTD into a mechanistic picture of complexin action, and comparisons across synapses of different species will aid and enrich our understanding of this fascinating regulatory protein.

## Author Contributions

Conceptualization: RW, DE, and JD. Methodology: RW, IB, DP, DS, MT, DE, HW, and JD. Investigation: RW, DP, MT, DS, ZL, IB, and JD. Writing: RW, HW, DE, and JD. Funding acquisition: DP, HW, DE, and JD.

## Conflict of Interest Statement

The authors declare that the research was conducted in the absence of any commercial or financial relationships that could be construed as a potential conflict of interest.

## Supplementary Material

TABLE S1 **Summary of axonal protein expression data for various transgenic animals. Quantification of axonal CPX-1::GFP expression for several transgenic animals used in this study.** Details of the imaging and quantification are described in the Methods. All average expression values were normalized to the full-length rescuing CPX-1::GFP transgenic (*tauIs90*), which is a multi-copy integrated array. In addition to the protein abundance for each transgenic, its qualitative ability to restore CPX-1 inhibitory function is also indicated (rescue quality). Importantly, the ability of CPX-1 variants to rescue the *cpx-1* null mutant generally did not correlate with their protein expression levels.
