# Supplementary material for: Evolutionary Divergence of the C-terminal Domain of Complexin Accounts for Functional Disparities between Vertebrate and Invertebrate Complexins
Source: Front Mol Neurosci. 2017 May 26;10:146. doi: 10.3389/fnmol.2017.00146 (PMC5445133; doi:10.3389/fnmol.2017.00146)
Supplement: TABLE S1 — Summary of axonal protein expression data for various transgenic animals. Quantification of axonal CPX-1::GFP expression for several transgenic animals used in this study. Details of the imaging and quantification are described in the Methods. All average expression values were normalized to the full-length rescuing CPX-1::GFP transgenic (tauIs90), which is a multi-copy integrated array. In addition to the protein abundance for each transgenic, its qualitative ability to restore CPX-1 inhibitory function is also indicated (rescue quality). Importantly, the ability of CPX-1 variants to rescue the cpx-1 null mutant generally did not correlate with their protein expression levels. [file Table_1.PDF]

Table 1 Axonal CPX Transgene Expression Levels

genotype	transgene	avgerage expression (norm)	SEM	N	rescue quality
tauIs90	high copy FL	1.00	0.097	21	good
tauIs141	Single copy FL	0.31	0.064	15	good
tauEx139	mCpx1	1.17	0.100	21	poor
tauEx192	mAH	1.74	0.157	8	good
tauEx211	mCH	1.61	0.231	20	good
tauEx115	mCTD	0.66	0.124	20	poor
tauEx299	CPX-W	0.93	0.156	21	good
tauEx354	CPX(Δ6)	0.77	0.045	24	poor
tauEx267	3xF/A	1.12	0.159	20	poor
tauEx304	3xF/I	1.34	0.141	21	intermed.
tauEx398	CPX(m1AR)	1.15	0.183	21	poor
tauEx379	CPX(m3AR)	1.20	0.177	21	intermed.
tauEx399	CPX(AR+AA)	0.77	0.098	19	poor
tauEx420	CPX(AR+4xA)	0.54	0.074	21	poor
