# Supplementary material for: Evolutionary Divergence of the C-terminal Domain of Complexin Accounts for Functional Disparities between Vertebrate and Invertebrate Complexins
Source: Front Mol Neurosci. 2017 May 26;10:146. doi: 10.3389/fnmol.2017.00146 (PMC5445133; doi:10.3389/fnmol.2017.00146)
Supplement: Supplementary file 2 [file Table_2.PDF]

Table 2 Species List

**Nematodes**	
Tt	*Trichuris trichiura*
Cb	*Caenorhabditis briggsae*
Ce	*Caenorhabditis elegans*
Sr	*Strongyloides ratti*
Bm	*Brugia malayi*
Ov	*Onchocerca volvulus*

**Vertebrates**	
Dr	*Danio rerio*
Pm	*Petromyzon marinus*
Xl	*Xenopus laevis*
Nj	*Narke japonica*
Gg	*Gallus gallus*
Hs	*Homo sapiens*
Rn	*Rattus norvegicus*
Mm	*Mus mus*

**Arthropods**	
Af	*Apis floria*
Tc	*Tribolium castaneum*
Dp	*Daphnia pulex*
Dm	*Drosophila melanogaster*
Sm	*Stegodyphus mimosarum*
Mo	*Metaseiulus occidentalis*
Mo	*Ixodes scapularis*
