# Supplementary material for: Evolutionary Divergence of the C-terminal Domain of Complexin Accounts for Functional Disparities between Vertebrate and Invertebrate Complexins
Source: Front Mol Neurosci. 2017 May 26;10:146. doi: 10.3389/fnmol.2017.00146 (PMC5445133; doi:10.3389/fnmol.2017.00146)
Supplement: Supplementary file 3 [file Table_3.PDF]

Table 3 Strain List

strain	genotype	transgene in *cpx-1(ok1152)*
JSD0291	tauIs90	CPX-1::GFP multi-copy
JSD0320	tauEx63	CPX-1(L117E V121V)::GFP
JSD0347	tauIs102	CPX-1(ΔCTD)::GFP
JSD0407	tauEx93	CPX-1::(Δ12)::GFP
JSD0438	tauIs141	CPX-1::GFP single-copy
JSD0511	tauEx115	CPX-1(Δ34+m1 last 34 CTD)::GFP
JSD0552	tauEx139	mCpx1::GFP
JSD0554	tauEx142	CPX-1(m1 NTD)::GFP
JSD0654	tauEx192	CPX-1(m1 AH)::GFP
JSD0669	tauEx211	CPX-1(m1 CH)::GFP
JSD0718	tauEx252	CPX-1(Δ12+m1 last 11 CTD)::GFP
JSD0747	tauEx267	CPX-1(3x F/A)::GFP
JSD0787	tauEx292	CPX-1(F137A)::GFP
JSD0812	tauEx299	CPX-1+W144::GFP
JSD0821	tauEx304	CPX-1(3x F/I)::GFP
JSD0903	tauEx354	CPX-1(Δ6)::GFP
JSD0904	tauEx355	CPX-1(Δ6+m7)::GFP
JSD0922	tauEx363	CPX-1(K143R)::GFP
JSD0923	tauEx364	CPX-1(K143A)::GFP
JSD0953	tauEx379	CPX-1(m3AR)::GFP
JSD0990	tauEx393	CPX-1 with m3AR -2 shift
JSD0995	tauEx398	CPX-1(m1AR)::GFP
JSD1006	tauEx399	CPX-1(AR+AA)::GFP
JSD1035	tauEx420	CPX-1(AR+AAAA)::GFP
JSD1098	tauIs53	CPX-1(K71A Y72A)::GFP

strain	genotype	other transgenes
JSD0110	tauIs55	CPX-1::GFP+mCherry::RAB-3 under Punc-129
JSD0469	tauEx102	SNB-1(DLV/AAA) in *snb-1(js104)*
JSD0538	tauEx133	UNC-64(LMDMD/5xA) in *unc-64(js115)*
JSD0819	tauEx302	mCpx1::GFP+mCherry::RAB-3 under Punc-129
JSD1034	tauEx419	HisCl channel under Punc-17

strain	genotype
CB1091	*unc-13(e1091)*
RB1367	*cpx-1(ok1552)*
NM467	*snb-1(md247)*
NM1081	*snb-1(js124)/dpy-11(e224) unc-68(r1158)*
CB246	*unc-64(e246)*
NM979	*unc-64(js115)/bli-5(e518)*
NM1968	*slo-1(js379)*
